# Peptide Transporter 1‐Mediated Dipeptide Transport Promotes Hepatocellular Carcinoma Metastasis by Activating MAP4K4/G3BP2 Signaling Axis

**DOI:** 10.1002/advs.202306671

**Published:** 2024-04-19

**Authors:** Feifeng Song, Zhentao Zhang, Weifeng Liu, Tong Xu, Xiaoping Hu, Qiyue Wang, Wanli Zhang, Luqi Ge, Chengwu Zhang, Qing Hu, Hui Qin, Song Zhang, Xinxin Ren, Weijiao Fan, Yiwen Zhang, Ping Huang

**Affiliations:** ^1^ Center for Clinical Pharmacy, Cancer Center Department of Pharmacy Zhejiang Provincial People's Hospital (Affiliated People's Hospital) Hangzhou Medical College Hangzhou 310014 China; ^2^ Key Laboratory of Endocrine Gland Diseases of Zhejiang Province Hangzhou 310014 China; ^3^ Zhejiang Provincial Clinical Research Center for malignant tumor Hangzhou 310014 China; ^4^ Department of Hepatobiliary and Pancreatic Surgery The Second Affiliated Hospital, Zhejiang University School of Medicine Hangzhou 310009 China; ^5^ Department of Hepatobiliary & Pancreatic Surgery and Minimally Invasion Surgery Zhejiang Provincial People's Hospital (Affiliated People's Hospital) Hangzhou Medical College Hangzhou 310014 China

**Keywords:** dipeptides, hepatocellular carcinoma, MAP4K4/G3BP2 signaling, metastasis, PEPT1, phosphorylation

## Abstract

Cancer metastasis is the leading cause of mortality in patients with hepatocellular carcinoma (HCC). To meet the rapid malignant growth and transformation, tumor cells dramatically increase the consumption of nutrients, such as amino acids. Peptide transporter 1 (PEPT1), a key transporter for small peptides, has been found to be an effective and energy‐saving intracellular source of amino acids that are required for the growth of tumor cells. Here, the role of PEPT1 in HCC metastasis and its underlying mechanisms is explored. PEPT1 is upregulated in HCC cells and tissues, and high PEPT1 expression is associated with poor prognosis in patients with HCC. PEPT1 overexpression dramatically promoted HCC cell migration, invasion, and lung metastasis, whereas its knockdown abolished these effects both in vitro and in vivo. Mechanistic analysis revealed that high PEPT1 expression increased cellular dipeptides in HCC cells that are responsible for activating the MAP4K4/G3BP2 signaling pathway, ultimately facilitating the phosphorylation of G3BP2 at Thr227 and enhancing HCC metastasis. Taken together, these findings suggest that PEPT1 acts as an oncogene in promoting HCC metastasis through dipeptide‐induced MAP4K4/G3BP2 signaling and that the PEPT1/MAP4K4/G3BP2 axis can serve as a promising therapeutic target for metastatic HCC.

## Graphical abstract

PEPT1 is upregulated in HCC and mediated the transport of dipeptides to enhance the epithelial and mesenchymal transformation of tumor cells by inducing MAP4K4‐dependent G3BP2 phosphorylation, thereby sustaining the survival of tumor cells and facilitating HCC metastasis.

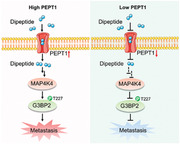

## 1 Introduction

Hepatocellular carcinoma (HCC) is one of the most fatal malignant tumors of the digestive system and the third leading cause of cancer‐related deaths worldwide.^[^ ^1^ ^]^ The high mortality rate is mainly due to high rates of recurrence or metastasis after surgical resection, local ablation, and liver transplantation.^[^ ^2^, ^3^, ^4^ ^]^ To date, despite multiple therapeutic options have been applied in patients with HCC to improve the survival, including chemotherapy, targeted therapy, and immunotherapy,^[^ ^5^, ^6^ ^]^ the overall treatment efficacy remains low, and there are no effective therapeutic methods for advanced or metastatic HCC.^[^ ^2^, ^7^ ^]^ Therefore, an in‐depth study on the characteristics of HCC metastasis is urgently required. It is vital to explore the key molecules and their underlying pathological mechanisms in HCC metastasis, which could be used to develop novel therapeutic strategies for HCC treatment.

Nutrient supplies, such as glucose and amino acids, are pivotal for tumor cells to maintain biological processes, such as protein synthesis, cell growth, and cell metabolism.^[^ ^8^ ^]^ To meet their rapid malignant growth and transformation, the consumption of nutrients is dramatically increased in tumor cells. As most of these molecules are hydrophilic, specific vehicles are needed for transmembrane transport. Accordingly, following augmented nutrient requirements, the expression and function of several transporters are enhanced to accelerate the cellular accumulation of nutrients. In mammals, the uptake of small peptides by proton‐coupled oligopeptide transporters (POTs) is an effective and energy‐saving intracellular source of amino acids.^[^ ^9^, ^10^ ^]^ POT family is also referred to as the solute carrier (SLC) family SLC15A, which contains four mammalian members: peptide transporter 1 (PEPT1) (encoded by *SLC15A1*), PEPT2 (encoded by *SLC15A2*), PHT1 (encoded by *SLC15A4*), and PHT2 (encoded by *SLC15A3*).

Among these transporters, PEPT1 is abundantly expressed in the intestine and is found in the liver, bile duct, pancreas, and placenta.^[^ ^11^, ^12^ ^]^ PEPT1 possesses sequence and substrate specificity similar to that of PEPT2 and mediates the uptake of dipeptides, tripeptides, and peptide‐like drugs in intestinal epithelial cells.^[^ ^11^, ^12^ ^]^ Accumulating evidence has shown that PEPT1 plays a critical role in the initiation and progression of inflammatory bowel disease,^[^ ^13^, ^14^, ^15^, ^16^ ^]^ but its function in tumors remains controversial. It was recently reported to be downregulated in colorectal cancer, and its transcriptional repression was mainly due to increased expression of DNMT1 and HDAC1.^[^ ^17^ ^]^ Nevertheless, other studies have demonstrated that PEPT1 expression is elevated in tumors and is associated with tumor growth and anti‐tumor drug absorption. For instance, PEPT1 expression was found to be increased in human colon tumor tissues, and high expression of PEPT1 promoted the proliferation and inhibited apoptosis of colonic epithelial cells by regulating the NF‐κB pathway.^[^ ^18^ ^]^ In pancreatic cancer, PEPT1 was markedly upregulated in pancreatic cell lines and patient‐derived xenografts, and silencing of PEPT1 inhibited colony formation and tumor growth.^[^ ^19^, ^20^ ^]^ Moreover, PEPT1 was found to be located in the mitochondrial membrane of human prostate cancer cells, where it induces tumor cell apoptosis by facilitating the transportation of melatonin to the mitochondria.^[^ ^21^ ^]^ Additionally, Guo et al. reported that PEPT1 expression is elevated in HCC cell lines and tissues, which could improve anti‐tumor efficacy by increasing the cellular accumulation of a tripeptide conjugated to doxorubicin.^[^ ^22^, ^23^ ^]^ However, no studies have clarified the role of PEPT1 and its clinical significance in HCC metastasis.

In this study, we found significant upregulation of PEPT1 in HCC cells and clinical tissues, which was associated with the poor prognosis of patients. Gain‐of‐function and loss‐of‐function experiments revealed that PEPT1 promoted HCC cell migration, invasion, and lung metastasis, whereas its knockdown abolished these effects in vitro and in vivo. Mechanistic analysis demonstrated that PEPT1 increases dipeptide accumulation in tumor cells. Augmented dipeptides induce the expression of MAP4K4 and promote the phosphorylation of G3BP2 at Thr227, leading to the activation of epithelial‐mesenchymal transition (EMT) signaling, which facilitates the metastasis of tumor cells. Overall, we elucidated a novel mechanism by which PEPT1‐mediated transportation of dipeptides activates MAP4K4/G3BP2 signaling to accelerate the metastasis of tumor cells. Our results revealed that the PEPT1/MAP4K4/G3BP2 signaling axis is essential for HCC growth and metastasis, and this mechanism may serve as a novel therapeutic target for clinical application in patients with metastatic HCC in the future.

## 2 Results

### 2.1 PEPT1 was Upregulated in HCC and Indicated Poor Prognosis

**Figure 1:**
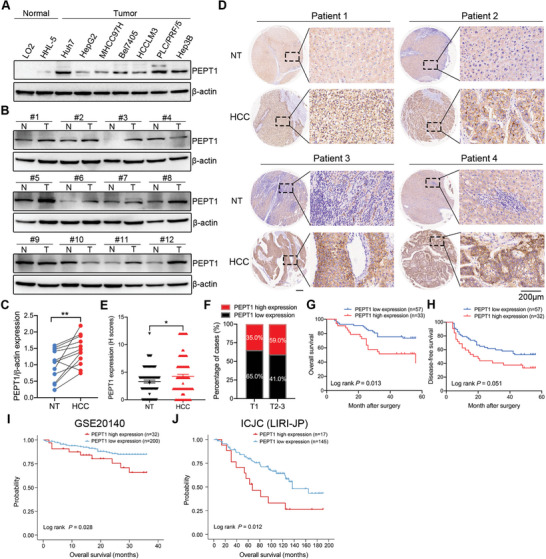
PEPT1 was upregulated in HCC and predicted poor clinical outcome. A) The protein expression levels of PEPT1 in normal hepatocytes and several HCC cell lines were detected by Western blot. B,C) PEPT1 protein levels in fresh HCC and adjacent nontumor tissues detection by Western blot (*n* = 12). D) Representative IHC images of PEPT1 in HCC tissue (*n* = 90) and corresponding normal tissue (*n* = 90). Scale bar, 200 µm. E) The corresponding IHC scores (H score) was compared on the right. F) Proportion of different expression levels of PEPT1 in HCC patients at different stages. G,H) Kaplan–Meier analysis of overall survival (OS) or disease‐free survival (DFS) curves after having assigned HCC patients to high/low of PEPT1 expression subgroups. I,J) Kaplan–Meier analysis of overall survival (OS) data from GSE20140 (I) and ICJC (LIRI‐JP) liver cancer database. ^*^ *P* < 0.05, ^**^ *P* < 0.01, and ^***^ *P* < 0.001.

To investigate the role of PEPT1 in HCC metastasis, we first analyzed the expression of PEPT1 in patients with HCC based on the UALCAN database. The data showed that PEPT1 transcript levels were significantly higher in HCC tissues than those in normal tissues (Figure S1A, Supporting Information). Moreover, the mRNA level of PEPT1 was found to be correlated with the clinical stage, pathological grade, and nodal metastasis of HCC tumor tissues (Figure S1B–D, Supporting Information). To further verify these results, the protein expression of PEPT1 in HCC cell lines and tissues was detected using Western blotting. Elevated PEPT1 expression was observed in all HCC cell lines (**Figure** **1**A). Consistent with cell expression, PEPT1 protein levels were markedly increased in HCC tissues compared to those in matched non‐tumor tissues in 11 out of 12 paired samples (Figure 1B,C). Higher PEPT1 protein levels in HCC tissues were confirmed via immunohistochemistry (IHC) (Figure 1D). Taken together, these results indicated that PEPT1 was upregulated in HCC.

To explore the clinical significance of PEPT1 in HCC progression, a tissue microarray (TMA) containing 90 primary HCCs and 90 paired non‐tumor tissues were determined via IHC and scored as previously reported.^[^ ^24^ ^]^ As shown in Figure 1D,E, the PEPT1 IHC score was notably higher in tumor tissues than that in the adjacent non‐tumor liver tissues. Further analysis of clinicopathological parameters and PEPT1 expression revealed that PEPT1 expression was significantly correlated with the pathological grade of the HCC samples (Figure 1F; Tables S1–S3, Supporting Information). Moreover, patients with HCC and high PEPT1 expression exhibited short overall survival rates and disease‐free survival rates (Figure 1G,H). Consistently, GEO (GSE20140) and ICGC (LIRI‐JP) data analysis also demonstrated an association between higher PEPT1 expression and shorter overall survival (Figure 1I,J). These data demonstrated that high PEPT1 expression was associated with HCC progression and patient prognosis.

### 2.2 PEPT1 Modulated HCC Cell Metastasis In Vitro and In Vivo

**Figure 2:**
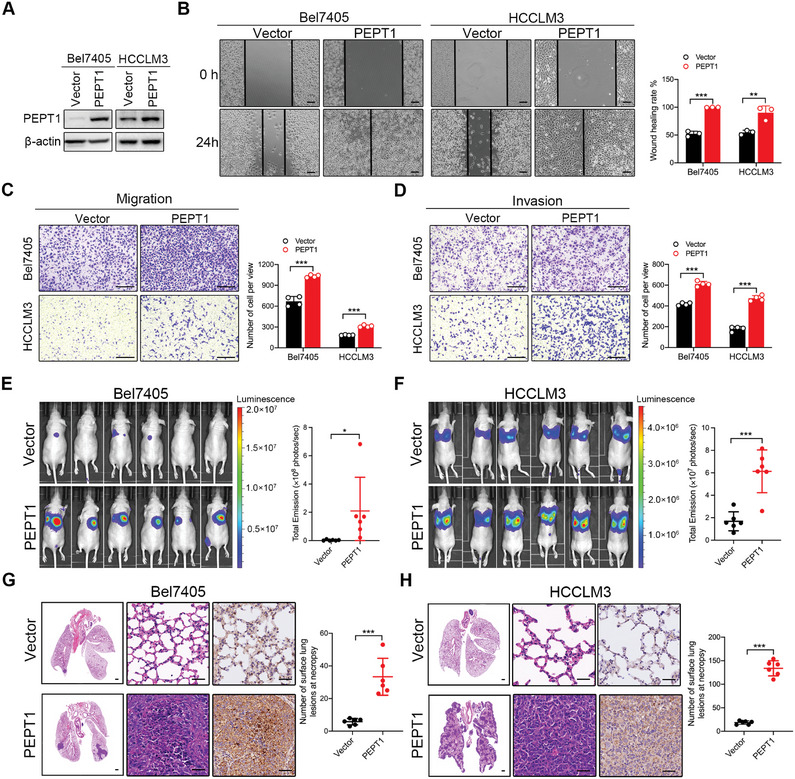
PEPT1 overexpression promoted HCC metastasis in vitro and in vivo. A) Bel7405 and HCCLM3 cells were infected with lentiviral particles expressing PEPT1 plasmid. The overexpression efficacy was detected by Western blot analysis. B) Representative images and quantification of the indicated cells in the wound‐healing migration assays. Scale bar, 100 µm. C,D) Representative images and quantification of the migration and invasion of the indicated cells in the Transwell assays. Scale bar, 250 µm. E,F) Representative images and quantification of the luciferase expression in pulmonary metastatic lesions. G,H) Representative images of HE and IHC staining of lungs resected from tail vein injection metastasis mouse model. Scale bar, 500 µm, 50 µm. The number of lung metastatic tumors in each group were determined. ^*^ *P* < 0.05, ^**^ *P* < 0.01, and ^***^ *P* < 0.001.

**Figure 3:**
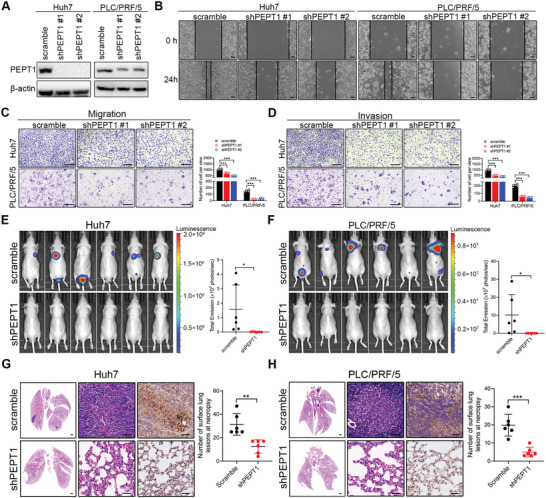
PEPT1 knockdown inhibited HCC metastasis in vitro and in vivo. A) Huh7 and PLC/PRF/5 cells were infected with lentiviral particles expressing shRNA targeting PEPT1. The knockdown efficacy was detected by Western blot analysis. B) Representative images and quantification of the indicated cells in the wound‐healing migration assays. Scale bar, 100 µm. C,D) Representative images and quantification of the migration and invasion of the indicated cells in the Transwell assays. Scale bar, 250 µm. E,F) Representative images and quantification of the luciferase expression in pulmonary metastatic lesions. G,H) Representative HE staining and IHC results of PEPT1 in lungs metastatic tumor resected from tail vein injection metastasis mouse model. Scale bar, 500 µm, 50 µm. The number of lung metastatic tumors in each group were determined. ^*^ *P* < 0.05, ^**^ *P* < 0.01, and ^***^ *P* < 0.001.

Considering the correlation between PEPT1 expression and HCC progression, we evaluated the effects of PEPT1 on the migratory and invasive capabilities of HCC cells in vitro. Stable cell lines with PEPT1 overexpression or knockdown were constructed in HCC cells with relatively low endogenous levels of PEPT1 (Bel7405 and HCCLM3) and relatively high endogenous levels of PEPT1 (Huh7 and PLC/PRF/5), respectively (Figure 1A). The efficiency of overexpression and knockdown was determined via Western blotting (**Figures** **2**A and **3**A). The ectopic expression of PEPT1 in Bel7405 and HCCLM3 cells dramatically enhanced their migration and invasion ability of HCC cells (Figure 2B–D). Conversely, knocking down PEPT1 in Huh7 and PLC/PRF/5 cells significantly decreased their migration and invasion capacities (Figure 3B–D).

The promoting effect of PEPT1 on the migration and invasion of HCC cells in vitro indicates its metastatic potential in vivo. To validate this hypothesis, luciferase‐labeled control and PEPT1‐overexpressed Bel7405 or HCCLM3 cells were injected into the tail vein of a lung metastatic nude mouse model. Luciferase signal intensities in the PEPT1 overexpressed group were much stronger than those in the control group (Figure 2E). Moreover, ectopic expression of PEPT1 resulted in a notably increased number of metastatic nodules and high PEPT1 expression in lung tissues (Figure 2G). A similar phenotype was observed in the same model injected with HCCLM3 cells (Figure 2F,H). Conversely, in the PEPT1 knockdown (Huh7 and PLC/PRF/5) groups, signal intensities, number of metastatic nodules, and PEPT1 expression in lung tissues decreased (Figure 3E–H). Taken together, these results illustrate that PEPT1 plays a vital role in facilitating HCC metastasis both in vitro and in vivo.

### 2.3 PEPT1 Promoted HCC Cell Metastasis via MAP4K4

**Figure 4:**
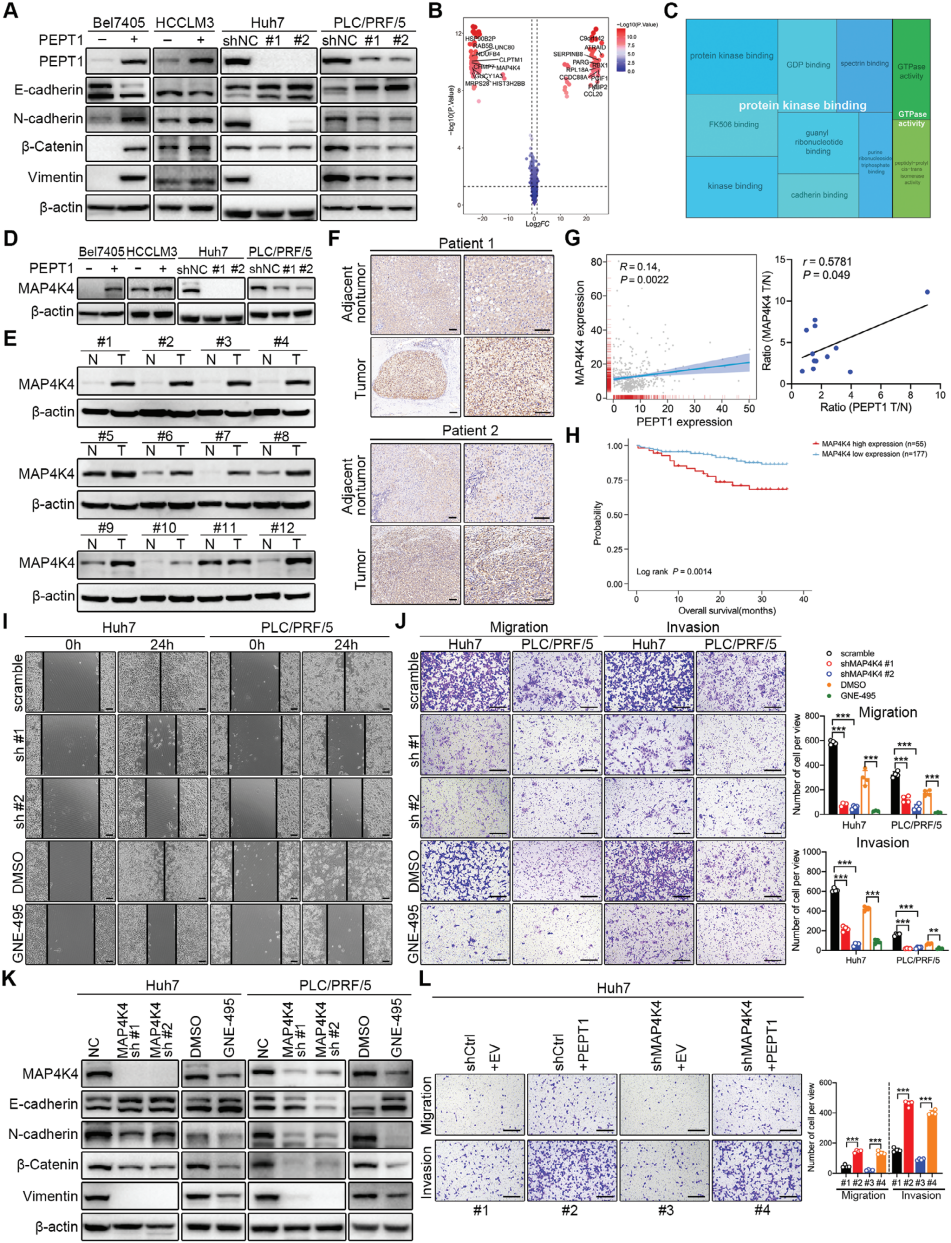
PEPT1‐mediated HCC metastasis was dependent on MAP4K4. A) Protein expression of EMT‐associated proteins in HCC cells with PEPT1 overexpression or knockdown. B) Volcano plot of all differential genes in Huh7 cells that stably express shRNA stargeting PEPT1 or scramble control. C) Go analysis showed that differentially expressed genes were mainly enriched in protein kinase binding. D) The protein expression of MAP4K4 in PEPT1‐overexpression or PEPT1‐silencing HCC cells was detected by Western blot analysis. E) MAP4K4 protein levels in fresh HCC and adjacent nontumor tissues detection by Western blot (*n* = 12). F) Representative IHC images of MAP4K4 in HCC tissue (*n* = 10) and corresponding normal tissue (*n* = 10). Scale bar, 100 µm. G) The correlation between PEPT1 and MAP4K4 was analyzed based on HCC date from the ICJC (LIRI‐JP) database (left) and Western blot results (right). H) Kaplan–Meier analysis of overall survival (OS) data from ICJC (LIRI‐JP) liver cancer database. I) Representative images and quantification of the indicated cells in the wound‐healing migration assays. Scale bar, 100 µm. J) Representative images and quantification of the migration and invasion of the indicated cells in the Transwell assays. Scale bar, 250 µm. K) Protein expression of EMT‐associated proteins in HCC cells with MAP4K4 knockdown. L) Representative images and quantification of the migration and invasion of the indicated cells in the Transwell assays. Scale bar, 250 µm. ^*^ *P* < 0.05, ^**^ *P* < 0.01, and ^***^ *P* < 0.001.

Given the pro‐metastatic role of PEPT1 in HCC, we detected changes in the expression of EMT‐associated protein in PEPT1 overexpressing and deficiency HCC cells using Western blotting. As shown in **Figure** **4**A, overexpression of PEPT1 downregulated the levels of epithelial proteins (e.g., E‐cadherin) and upregulated the levels of mesenchymal‐associated proteins (e.g., N‐cadherin, β‐catenin, and vimentin), whereas knockdown of PEPT1 restored the expression of these proteins. To further elucidate the mechanism of PEPT1‐mediated HCC metastasis, we performed a proteomic analysis using a vector and PEPT1 silencing Huh7 cells. A total of 156 differentially expressed proteins were identified, and the top 10 upregulated and downregulated proteins are shown in Figure 4B. Functional enrichment analysis revealed that the downregulated proteins were associated with protein kinase binding (Figure 4C). Among them, mitogen‐activated protein kinase 4 (MAP4K4) was identified, which is a serine/threonine protein kinase and has been reported to play a critical role in inflammation, cardiovascular diseases, and cancer.^[^ ^25^, ^26^, ^27^ ^]^ Importantly, MAP4K4 contributes to tumorigenesis and tumor progression.^[^ ^25^, ^28^ ^]^ To validate the proteomic results, we determined the protein levels of MAP4K4 in stable PEPT1‐overexpressing and PEPT1‐knockdown cell lines. The results showed that the ectopic expression of PEPT1 significantly increased the expression of MAP4K4 protein, whereas silencing of PEPT1 markedly decreased the expression of MAP4K4 protein (Figure 4D). Moreover, immunoblot analysis demonstrated that the protein level of MAP4K4 was also notably increased in HCC cells compared to that in normal hepatocytes (Figure S2A, Supporting Information). Next, we detected the protein expression of MAP4K4 on the same set of HCC tissues and found that MAP4K4 expression was considerably higher in HCC tissues than that in matched non‐tumor tissues in the 12 paired samples (Figure 4E). Similar results were obtained for HCC tissues via IHC staining (Figure 4F). In addition, correlation analysis based on ICGC (LIRI‐JP) data and Western blot results of PEPT1 and MAP4K4 showed that PEPT1 was significantly associated with MAP4K4 in HCC tissues (Figure 4G). Further prognosis analysis based on ICGC database showed that HCC patients with higher MAP4K4 expression suggested worse overall survival (Figure 4H).

To explore whether PEPT1‐mediated HCC metastasis is dependent on MAP4K4, we silenced endogenous MAP4K4 in Huh7 and PLC/PRF/5 cells by transfecting them with lentiviral MAP4K4 shRNA. The knockdown efficacy was examined using Western blotting (Figure 4K). Wound healing and Transwell assays demonstrated that MAP4K4 depletion suppressed cell migration and invasion compared to the control group (Figure 4I,J). Moreover, the expression of EMT‐associated proteins was inhibited (Figure 4K). To further confirm that MAP4K4 inhibition abrogated PEPT1‐mediated HCC metastasis, we treated Huh7 and PLC/PRF/5 cells with a specific MAP4K4 inhibitor, GNE‐495. Cell Counting Kit 8 (CCK8) and immunoblot assays showed that the expression of MAP4K4 was dramatically diminished in cells treated with GNE‐495, without influencing cell growth (Figure S2B,C, Supporting Information). The suppressive effect of GNE‐495 on MAP4K4 expression inhibited cell migration, invasion, and EMT (Figure 4I–K). In addition, we overexpressed PEPT1 in MAP4K4‐silenced Huh7 and PLC/PRF/5 cells (Figure S2D,F, Supporting Information). We found that restoration of PEPT1 expression rescued the decreased cell migratory and invasive abilities upon MAP4K4 depletion (Figure 4L; Figure S2E, Supporting Information). Taken together, these findings suggest that MAP4K4 is required for the metastasis‐promoting effects of PEPT1.

### 2.4 MAP4K4 Interacted and Colocalized with G3BP2 in HCC Cells

**Figure 5:**
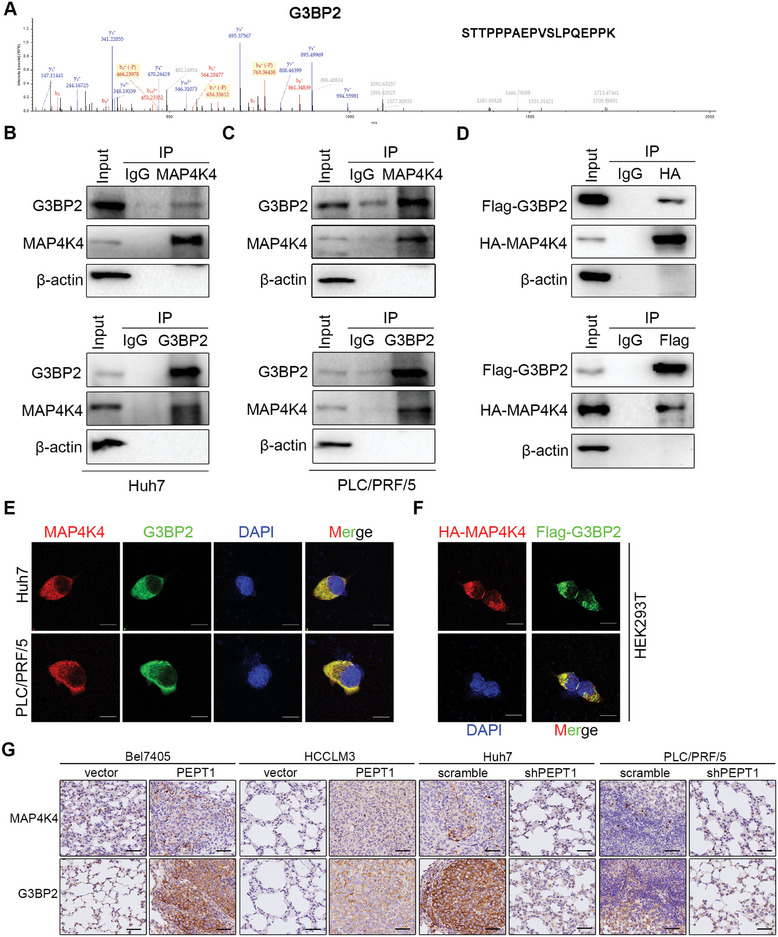
MAP4K4 directly binds to G3BP2 in HCC. A) Phosphorylated proteomics analysis identified G3BP2 in the binding protein pool. B,C) Endogenous interaction between MAP4K4 and G3BP2 was determined using co‐IP with anti‐MAP4K4 or anti‐G3BP2 antibodies in Huh7 and PLC/PRF/5 cells. D) Exogenous interaction between MAP4K4 and G3BP2 was determined using co‐IP with anti‐Flag or anti‐HA antibodies in HEK 293T cells co‐transfected with Flag‐G3BP2 and HA‐MAP4K4. E) Immunofluorescence staining showing colocalization of endogenous MAP4K4 (red) and G3BP2 (green) in Huh7 and PLC/PRF/5 cells. The nucleus is labeled by DAPI (blue). Scale bar: 20 µm. F) Immunofluorescence staining showing colocalization of exogenous HA‐MAP4K4 (red) and Flag‐G3BP2 (green) in HEK293T cells. The nucleus is labeled by DAPI (blue). Scale bar: 20 µm. G) Representative IHC images for MAP4K4 and G3BP2 in pulmonary metastatic lesions of nude mice developed by PEPT1‐knockdown or PEPT1‐overexpression HCC cells. Scale bar, 500 µm, 50 µm.

Considering that MAP4K4 is a serine/threonine protein kinase, we performed a phosphorylated proteomic analysis to identify the proteins that interact with MAP4K4. Approximately 1595 differentiated proteins were identified and five proteins might potentially interact with MAP4K4 (Table S4, Supporting Information). Interestingly, the Ras‐GTPase‐activating protein (SH3 domain)‐binding protein 2 (G3BP2) was found in the substrate pool (**Figure** **5**A). G3BP2 is an important molecule mainly distributed in the cytoplasm and is critical for signaling transduction, cell differentiation, and proliferation.^[^ ^29^ ^]^ In particular, accumulating evidence suggests that G3BP2 participates in the initiation and progression of various tumors.^[^ ^30^ ^]^ However, the precise role of G3BP2 in HCC progression remains unclear. A co‐immunoprecipitation assay (co‐IP) was performed to confirm the interaction between MAP4K4 and G3BP2 with endogenous proteins in HCC cells or exogenous proteins in HEK392T cells transfected with HA‐MAP4K4 and Flag‐G3BP2. The interaction between MAP4K4 and G3BP2 was detected using Western blotting (Figure 5B–D). To further validate this result, we detected the colocalization of endogenous MAP4K4 and G3BP2 in Huh7 and PLC/PRF/5 cells and exogenous MAP4K4 and G3BP2 in HEK293T cells using immunofluorescence and laser confocal microscope (Figure 5E,F). Moreover, lung metastatic nude mice with Bel7405 and HCCLM3 cells overexpressing PEPT1 displayed stronger staining for MAP4K4 and G3BP2 than the control group, whereas the staining of MAP4K4 and G3BP2 in PEPT1 knockdown mice was reversed (Figure 5G). Taken together, these results suggest that G3BP2 acts as a MAP4K4‐binding protein in HCC cells.

### 2.5 MAP4K4 Phosphorylated G3BP2 at T227 to Regulate HCC Cell Metastasis

**Figure 6:**
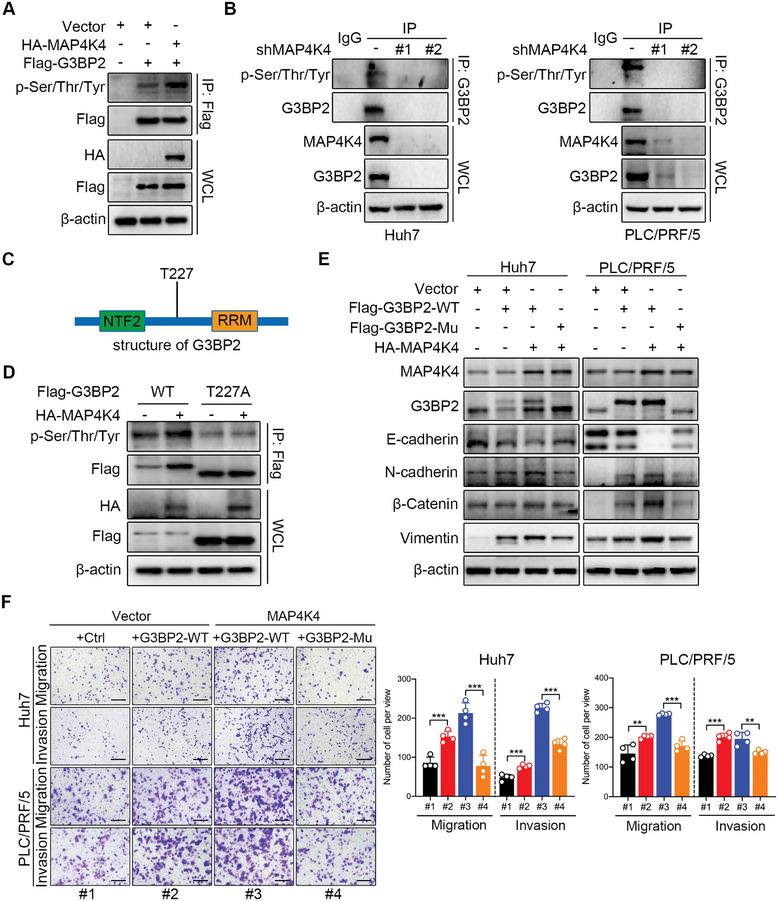
MAP4K4‐mediated phosphorylation at T227 is required for the function of G3BP2 in HCC metastasis. A) HEK293T cells were transfected with vectors, Flag‐G3BP2 along with HA‐MAP4K4. The cell extracts were used to immunoprecipitated Flag‐G3BP2 and blotted with anti‐p‐Ser/Thr/Tyr and anti‐Flag antibodies. The whole‐cell lysate (WCL) was blotted with anti‐HA, anti‐Flag, and anti‐β‐actin antibodies. B) The MAP4K4 knockdown Huh7 and PLC/PRF/5 extracts were used to immunoprecipitated G3BP2 and blotted with anti‐p‐Ser/Thr/Tyr and anti‐G3BP2 antibodies. The WCL was blotted with anti‐MAP4K4, anti‐G3BP2, and anti‐β‐actin antibodies. C) Schematic diagram of G3BP2 structure and phosphorylation sites. D) HEK293T cells were transfected with Flag‐G3BP2 (WT), Flag‐G3BP2 (T227A), or HA‐MAP4K4 as the indicated combinations. The cell extracts were used to immunoprecipitated Flag‐G3BP2 and blotted with anti‐p‐Ser/Thr/Tyr and anti‐Flag antibodies. The WCL was blotted with anti‐HA, anti‐Flag and anti‐β‐actin antibodies. E) Huh7 and PLC/PRF/5 cells were transfected with vector, G3BP2 (WT) or G3BP2 (T227A), and the protein expression of MAP4K4, G3BP2, EMT‐associated proteins were detected by Western blot. F) Representative images and quantification of the migration and invasion of the indicated cells in the Transwell assays. Scale bar, 250 µm. ^*^ *P* < 0.05, ^**^ *P* < 0.01, and ^***^ *P* < 0.001.

However, the substrates of MAP4K4, a serine/threonine kinase, remain unknown. A recent study reported that the MAP4K4‐mediated phosphorylation of mixed‐lineage kinase 3 (MLK3) promotes pancreatic cancer.^[^ ^28^ ^]^ However, in our phosphorylation proteomic analysis, MLK3 was not detected in the interacting protein pool. Thus, we speculated that MAP4K4 directly phosphorylates G3BP2. To verify this hypothesis, we transfected HEK293T cells with exogenous HA‐MAP4K4 and Flag‐G3BP2 and conducted an immunoprecipitation (IP) assay to detect the phosphorylated level of G3BP2 using a p‐Ser/Thr/Tyr antibody. The result showed that MAP4K4 increased the phosphorylation of G3BP2 (**Figure** **6**A). Moreover, the downregulation of MAP4K4 reduced the level of phosphorylated G3BP2 (Figure 6B). The phosphorylated site of G3BP2 was identified at T227 based on the phosphorylated proteomic data (Figure 6C). To confirm this result, we mutated threonine (T) at position 227 to alanine (A) to establish a G3BP2 mutant (T227A) and transfected it with HA‐MAP4K4 into HEK293T cells to evaluate the phosphorylation of G3BP2. Phosphorylated G3BP2 expression decreases in the T227A mutant in the absence of HA‐MAP4K4. However, MAP4K4 overexpression increased the phosphorylation levels of the T227A mutant (Figure 6D). These data indicated that MAP4K4 phosphorylates G3BP2 at T227. Next, we investigated whether T227 phosphorylation influenced HCC metastasis in Huh7 and PLC/PRF/5 cells transfected with exogenous wild‐type G3BP2 (Flag‐G3BP2‐WT) or T227 mutant G3BP2 (Flag‐G3BP2‐Mu). Western blotting showed that the overexpression of MAP4K4 and G3BP2 significantly enhanced the mesenchymal protein expression of EMT compared to the G3BP2 or vector group. However, mutation of G3BP2 at T227 dramatically downregulated EMT (Figure 6E). Consistent with these results, the Transwell assay showed that overexpression of MAP4K4 and G3BP2 notably promoted the migration and invasion of Huh7 and PLC/PRF/5 cells, which was impaired by the G3BP2 T227 mutant (Figure 6F).

**Figure 7:**
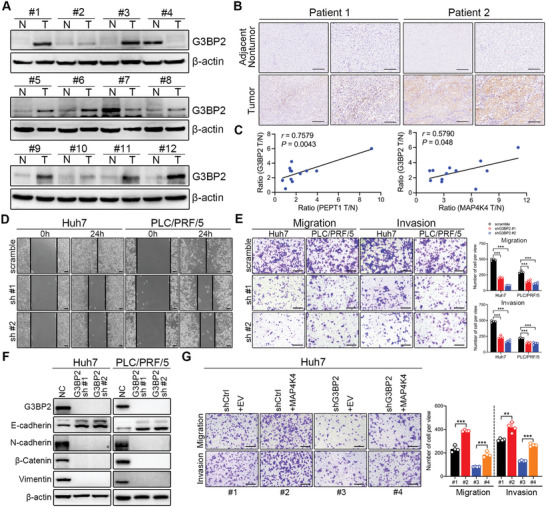
G3BP2 was upregulated in HCC and influenced cell migration and invasion. A) G3BP2 protein levels in fresh HCC and adjacent nontumor tissues detected by Western blot (*n* = 12). B) Representative IHC images of G3BP2 in HCC tissue (*n* = 10) and corresponding normal tissue (*n* = 10). Scale bar, 100 µm. C) The correlation between PEPT1 and G3BP2 (left), and MAP4K4 and G3BP2 (right) was analyzed based on the Western blot results. D) Representative images and quantification of the indicated cells in the wound‐healing migration assays. Scale bar, 100 µm. E) Representative images and quantification of the migration and invasion of the indicated cells in the Transwell assays. Scale bar, 250 µm. F) Protein expression of EMT‐associated proteins in HCC cells with G3BP2 knockdown. G) Representative images and quantification of the migration and invasion of the indicated cells in the Transwell assays. Scale bar, 250 µm. ^*^ *P* < 0.05, ^**^ *P* < 0.01, and ^***^ *P* < 0.001.

Considering that G3BP2 participates in tumor initiation and progression, we also investigated the role of G3BP2 in HCC metastasis. The protein expression of G3BP2 was markedly higher in HCC cells than that in normal hepatocytes (Figure S3A, Supporting Information). Moreover, G3BP2 was notably upregulated in HCC tissues compared to that in matched non‐tumor tissues in 10 of the 12 paired samples (**Figure** **7**A). Consistent with this result, IHC staining revealed elevated G3BP2 expression in the HCC tissues (Figure 7B). Further correlation analysis based on Western blot results showed that PEPT1 and MAP4K4 was significantly associated with G3BP2 (Figure 7C). Additionally, we performed wound healing and Transwell assays to investigate the effect of G3BP2 on HCC metastasis. The results showed that silencing of G3BP2 suppressed the wound healing, migration, and invasion of Huh7 and PLC/PRF/5 cells (Figure 7D,E). Furthermore, the levels of EMT‐associated proteins were reduced in the G3BP2‐knockdown group compared with those in the control group (Figure 7F). To further verify that G3BP2 was required for MAP4K4‐mediated HCC metastasis, we transfected G3BP2 silencing Huh7 and PLC/PRF/5 cells with HA‐MAP4K4. The overexpression efficacy was validated via Western blotting (Figure S3B, Supporting Information). Transwell assays showed that G3BP2 knockdown inhibited the migration and invasion of Huh7 and PLC/PRF/5 cells, whereas it was rescued by MAP4K4 overexpression but not restored by mutant G3BP2 (Figure 7G; Figure S3C–G, Supporting Information). These results suggest that PEPT1/MAP4K4 mediated HCC metastasis by regulating the expression and T227 phosphorylation of G3BP2.

### 2.6 PEPT1‐Mediated Dipeptide Transport was Required for MAP4K4/G3BP2 Axis Activation in HCC Metastasis

**Figure 8:**
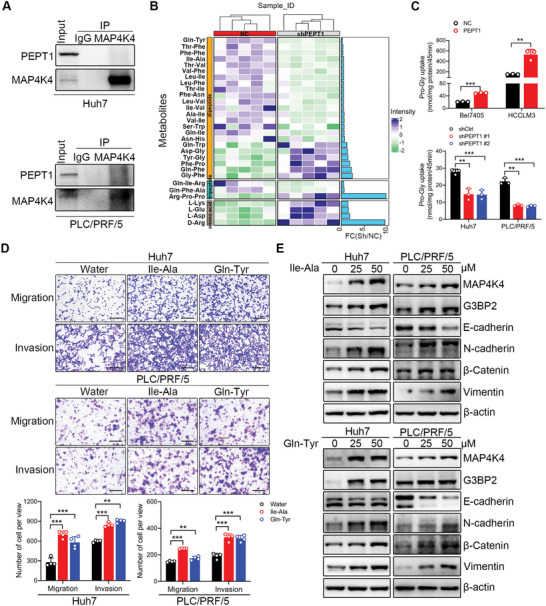
PEPT1‐mediated dipeptides transport was essential for activating MAP4K4/G3BP2 axis. A) Endogenous interaction between PEPT1 and MAP4K4 was determined using co‐IP with anti‐MAP4K4 antibodies in Huh7 and PLC/PRF/5 cells. B) Metabolomics analysis identified dipeptides downregulated in Huh7 cells with stably PEPT1 knockdown. C) The uptake of Pro‐Gly in HCC cells with PEPT1 overexpressing and knockdown. D) Representative images and quantification of the migration and invasion of the indicated cells in the Transwell assays. Scale bar, 250 µm. E) Huh7 and PLC/PRF/5 cells were incubated with the indicated concentrations of Ile‐Ala or Gln‐Tyr for 24 hours, and the protein expression of MAP4K4, G3BP2, EMT‐associated proteins were detected by Western blot. Error bars indicate means ± SD. ^*^ *P* < 0.05, ^**^ *P* < 0.01, and ^***^ *P* < 0.001.

Given that PEPT1 mediates HCC metastasis through MAP4K4 and that MAP4K4 interacts with G3BP2 to facilitate HCC metastasis, we speculated that PEPT1 may directly regulate MAP4K4. To verify this, we performed a co‐IP assay and found that PEPT1 did not interact with MAP4K4 (**Figure** **8**A). It was reported that PEPT1 functions as a transporter of dipeptides and tripeptides. Thus, we conducted metabolomics to explore the metabolite changes in Huh7 cells with PEPT1 knockdown. The result showed that PEPT1 silencing abrogated the transport of most dipeptides (Figure 8B; Figure S4, Supporting Information). To verify this result, we conducted an uptake study in stably transfected PEPT1 overexpression and knockdown cell lines. As shown in Figure 8C, the uptake of the PEPT1 substrate Pro‐Gly was significantly increased in Bel7405 and HCCLM3 cells overexpressing PEPT1. However, the knockdown of PEPT1 in Huh7 and PLC/PRF/5 cells clearly inhibited the uptake of Pro‐Gly (Figure 8C). To further analyze whether PEPT1‐mediated dipeptide transportation influences HCC cell metastasis, we treated Huh7 and PLC/PRF/5 cells with Ile‐Ala or Gln‐Tyr. Transwell assays showed that the dipeptides (Ile‐Ala and Gln‐Tyr) markedly enhanced HCC cell migration and invasion (Figure 8D). Moreover, elevated expression of MAP4K4 and G3BP2 was observed in Huh7 and PLC/PRF/5 cells treated with different concentrations of the dipeptides (Ile‐Ala or Gln‐Tyr) (Figure 8E). Additionally, EMT signaling was activated in HCC cells treated with the dipeptides (Figure 8E). These data indicated that PEPT1 activates MAP4K4/G3BP2 signaling to accelerate HCC metastasis by transporting dipeptides.

### 2.7 PEPT1/MAP4K4/G3BP2 Signaling Axis Facilitates HCC Cell Metastasis

**Figure 9:**
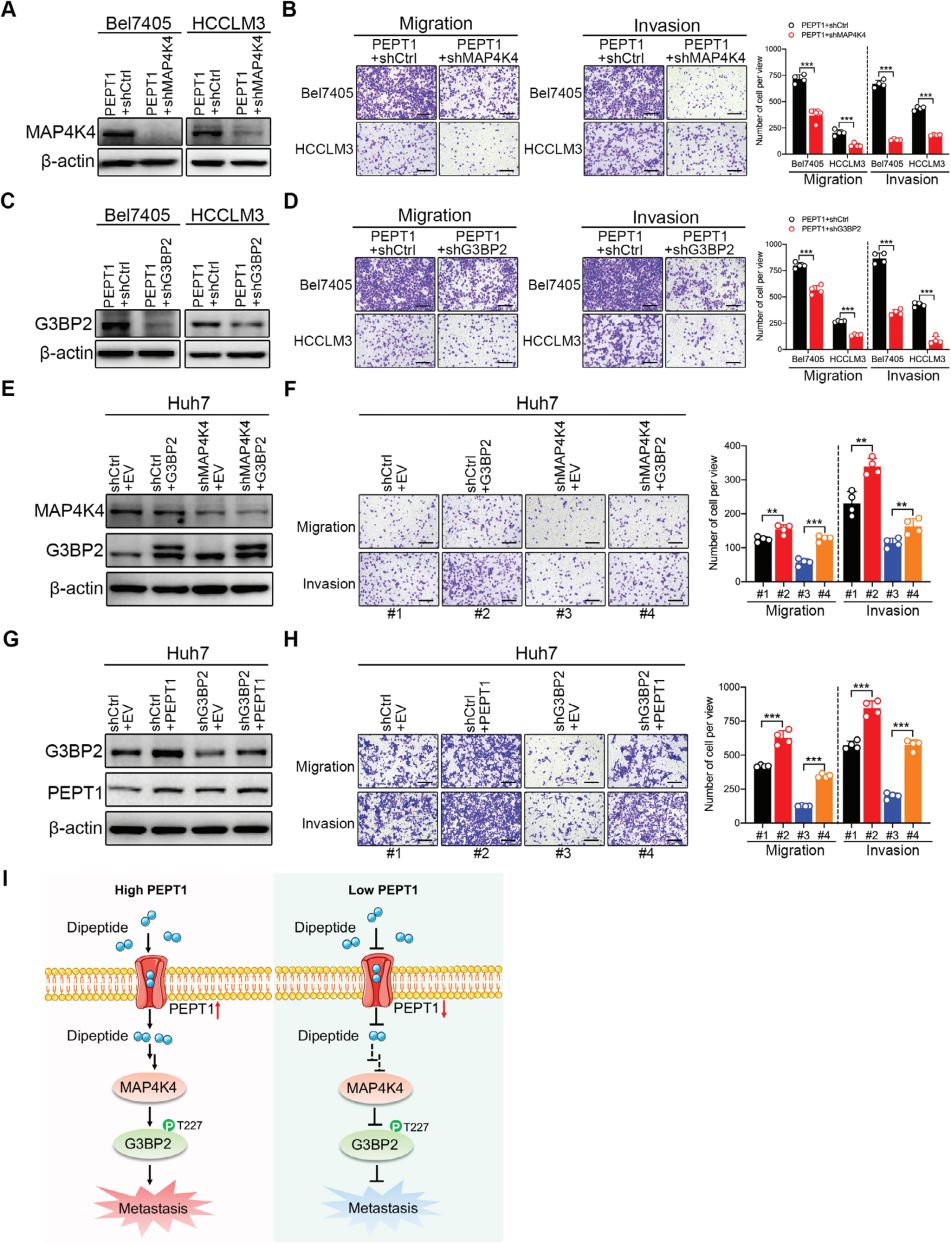
PEPT1/MAP4K4/G3BP2 signaling axis promoted HCC metastasis. A,C) Western blot analysis showed that the knockdown efficacy of MAP4K4 or G3BP2 in Bel7405 and HCCLM3 cells with PEPT1 overexpressing. B,D) Transwell assays revealed that migration and invasion ability was abrogated in Bel7405 and HCCLM3 cells with MAP4K4 or G3BP2 knockdown compared with the control group. Scale bar, 250 µm. E,G) Western blot analysis showed that the overexpression efficacy of G3BP2 (or PEPT1) in Huh7 cells with MAP4K4 (or G3BP2) knockdown. F,H) Inhibited migration and invasion of Huh7 cells with MAP4K4 (or G3BP2) knocked down was rescued by the overexpression of G3BP2 (or PEPT1). Left, representative images of Transwell assays, scale bar, 250 µm. Right, statistical analysis of Transwell assays. I) A schematic diagram of the PEPT1/MAP4K4/G3BP2 regulatory signaling axis that facilitates HCC metastasis.

To further confirm the critical role of the PEPT1/MAP4K4/G3BP2 signaling axis in HCC metastasis, we explored the effect of the interaction between one protein and the other two proteins. To confirm that MAP4K4/G3BP2 mediated PEPT1‐facilitated HCC metastasis, knockdown of either MAP4K4 or G3BP2 was performed in PEPT1‐overexpressing Bel7405 and HCCLM3 cells. The results showed that MAP4K4 and G3BP2 protein levels were significantly reduced (**Figure** **9**A,C), and cell migration and invasion were abrogated (Figure 9B,D). Conversely, overexpression of MAP4K4 or G3BP2 in PEPT1‐knockdown Huh7 and PLC/PRF/5 cells significantly eliminated the decrease in cell migration and invasion upon PEPT1 knockdown (Figure S5, Supporting Information). Considering that PEPT1 overexpression dramatically restored MAP4K4‐knockdown‐induced metastatic suppression (Figure 4J), we overexpressed G3BP2 in MAP4K4‐knockdown cells. Transwell assays showed that the overexpression of G3BP2 promoted metastasis and functionally rescued the reduction in cell migration and invasion induced by MAP4K4 silencing (Figure 9E,F; Figure S6A,B, Supporting Information). Given that MAP4K4 overexpression functionally reversed the metastatic suppression upon G3BP2 knockdown (Figure 7F), we investigated the effect of PEPT1 overexpression in G3BP2‐knockdown cells. The results showed that the induced expression of PEPT1 reversed G3BP2 expression (Figure 9G) and significantly eliminated the metastasis‐suppressive function upon G3BP2 silencing (Figure 9H; Figure S6C,D, Supporting Information). Taken together, our results suggest that the PEPT1/MAP4K4/G3BP2 signaling axis plays an indispensable role in accelerating HCC metastasis.

## 3 Discussion

PEPT1 has been reported to play a role in the progression of various tumors;^[^ ^19^, ^20^, ^21^, ^31^ ^]^ however, the underlying mechanism remains largely unknown. In this study, we demonstrated that PEPT1 was upregulated in HCC and mediated the transport of dipeptides to enhance the epithelial and mesenchymal transformation of tumor cells by inducing MAP4K4‐dependent G3BP2 phosphorylation, thereby sustaining the survival of tumor cells and facilitating HCC metastasis (Figure 9I).

PEPT1 and other POT family members are responsible for transporting dipeptides and tripeptides and are critical for maintaining the intracellular and extracellular homeostasis of nutrients. Several studies have demonstrated the critical role of PEPT1 in innate immunity, and the upregulation of PEPT1 occurs in patients with inflammatory bowel disease and short bowel syndrome.^[^ ^12^, ^32^ ^]^ As for cancer, studies have revealed an oncogenic role of PEPT1 in several cancers, including colon, pancreatic, and prostate cancers.^[^ ^18^, ^19^, ^20^, ^21^ ^]^ In addition, PEPT1 was reported to be upregulated in HCC, which is responsible for transporting a tripeptide‐conjugated doxorubicin to enhance the anti‐tumor efficacy.^[^ ^22^, ^23^ ^]^ However, the exact function and underlying mechanism of PEPT1 in HCC, especially in HCC metastasis, remain largely unknown. Herein, we demonstrated that PEPT1 was upregulated in HCC tissues and that high PEPT1 expression was significantly associated with short survival time in patients with HCC. Further analysis revealed that the overexpression of PEPT1 promoted in vitro HCC cell migration and invasion and facilitated tumor metastasis in vivo, whereas PEPT1 knockdown strikingly inhibited HCC metastasis. Mechanistically, PEPT1 induces EMT signaling by activating MAP4K4, thereby promoting HCC cell metastasis. Although PEPT1‐mediated HCC metastasis is dependent on MAP4K4, our co‐IP results revealed no direct interactions between PEPT1 and MAP4K4. A previous study demonstrated that PEPT2 promotes the absorption of dipeptide species in chronic myelogenous leukemia stem cells, activating p38MAPK‐mediated phosphorylation of Smad3, thereby maintaining stem cell activity.^[^ ^33^ ^]^ Considering that PEPT1 has a similar sequence and substrate as PEPT2, we examined the metabolic changes in PEPT1‐knockdown HCC cells. Interestingly, we found that most of the dipeptides dramatically decreased in HCC cells with PEPT1 silencing, and the amino acids increased accordingly. Moreover, elevated concentrations of dipeptides in HCC cells can induce the expression of MAP4K4, thereby accelerating tumor cell metastasis. Nevertheless, further investigations are needed to precisely elucidate how HCC tumor cells sense and utilize dipeptides to regulate MAP4K4.

Mitogen‐activated protein (MAP) kinases are a family of protein serine/threonine kinases that participate in transmitting the signals from extracellular to intracellular components. MAPKs are commonly activated by phosphorylation cascades and subsequently phosphorylate a series of effectors that regulate various cellular processes, including embryogenesis, cell differentiation, cell proliferation, and cell death.^[^ ^34^, ^35^ ^]^ MAP4K4 is a germinal center protein kinase belonging to the mammalian STE20/MAP4K family, which plays a role in activating the JNK/SAPK pathway.^[^ ^36^ ^]^ Numerous studies have implicated a crucial role of MAP4K4 in the regulation of the initiation and progression of multiple cancers. For instance, it has been shown that MAP4K4 is highly expressed in pancreatic cancer and its activation of mixed lineage kinase 3 promotes pancreatic tumorigenesis.^[^ ^28^ ^]^ Moreover, MAP4K4 has been shown to facilitate cell migration and invasion by diminishing the cleavage of N‐cadherin^[^ ^25^ ^]^ or activating c‐Jun N‐terminal kinase signaling in human ovarian cancer^[^ ^37^ ^]^ or inducing SOX6‐mediated autophagy and cisplatin resistance in human cervical cancer cells.^[^ ^38^ ^]^ Additionally, elevated protein levels of MAP4K4 have been found in human HCC tissues, and its silencing can significantly inhibit in vitro cell proliferation and xenograft tumor growth in vivo.^[^ ^36^ ^]^ Similarly, we found that the protein expression of MAP4K4 was upregulated in HCC cells and tissues, and its downregulation inhibited tumor cell migration and invasion. Mechanistically, activated MAP4K4 phosphorylates G3BP2 at T227, thereby promoting EMT signaling and subsequent tumor cell metastasis. Importantly, our findings, along with those of other researchers, revealed that a MAP4K4 inhibitor, GNE‐495, impedes hepatocellular cancer cell migration and invasion, indicating that MAP4K4‐targeting therapies may alleviate HCC progression.

G3BP2 belongs to the G3BP protein family and has conventionally been regarded as a key component of stress granules.^[^ ^39^ ^]^ G3BPs are found in many types of different cancers and participate in regulating various tumor initiation and progression‐related signaling pathways, such as Ras, mTOR, NF‐κB, and EMT signaling pathways.^[^ ^40^, ^41^, ^42^ ^]^ Although mounting evidence has revealed the critical role of G3BPs in the pathology of various tumors, the role of G3BP2 in cancer growth and metastasis remains controversial. The reduction or loss of G3BP2 expression contributes to induce EMT and promote breast tumor metastasis.^[^ ^42^, ^43^ ^]^ In contrast to its intensive role in tumor development, numerous studies have shown that the upregulation of G3BP2 is significantly correlated with unfavorable outcomes in colorectal cancer and esophageal squamous cell cancer.^[^ ^44^, ^45^ ^]^ However, the function and underlying molecular mechanisms of action of G3BP2 in HCC metastasis remain largely unknown. Here, we demonstrated that G3BP2 was upregulated in HCC cells and tissues and was phosphorylated by MAP4K4 at T227. Moreover, MAP4K4‐mediated T227 phosphorylation is essential for enhancing HCC metastasis because non‐phosphorylatable G3BP2‐T227A fails to induce EMT signaling, tumor cell migration, and invasion. Although many phosphorylated residues have been identified in G3BPs, such as S149^[^ ^46^ ^]^ and T226,^[^ ^44^ ^]^ the residues phosphorylated by G3BP2 in HCC and their corresponding influences remain unknown. To the best of our knowledge, this is the first study to report the role of G3BP2 in HCC metastasis and to identify a new phosphorylation site for G3BP2 at T227.

To better understand the critical role of the PEPT1/MAP4K4/G3BP2 signaling axis in facilitating HCC metastasis, a series of rescue experiments were conducted. Our data showed that silencing either MAP4K4 or G3BP2 significantly attenuated the enhanced effect of PEPT1 overexpression on HCC cell metastasis. In addition, ectopic overexpression of PEPT1 and MAP4K4 markedly restored the suppressive effects of G3BP2 knockdown on HCC cell migration. Moreover, the staining of PEPT1, MAP4K4, and G3BP2 in tumor and non‐tumor tissues exhibited the same pattern in the lung metastasis mouse model, which was also in accordance with in vitro functional assays.

In summary, our results reveal a novel regulatory signaling axis, PEPT1/MAP4K4/G3BP2, which is critical for inducing tumor metastasis in HCC, and provide a promising therapeutic target and prognostic indicator for patients with metastatic HCC.

## 4 Experimental Section

### Cell Lines and Cell Culture

Human HCC cell lines (Bel‐7405, HCCLM3, HepG2, Hep3B, Huh7, MHCC‐97H, and PLC/PRF/5), normal hepatic cells (LO2 and HHL‐5), and HEK293T cells were obtained from Fenghui Biotechnology Co., Ltd. (Hunan, China). Bel‐7405 and LO2 cells were cultured in RPMI‐1640 (HyClone). HCCLM3, HepG2, Hep3B, Huh7, MHCC‐97H, PLC/PRF/5, HHL‐5, and HEK293T cells were cultured in DMEM (HyClone). All media were supplemented with 10% fetal bovine serum (FBS, Gibco) and 1% penicillin/streptomycin (HyClone). All the cells were maintained in a humidified incubator containing 5% CO_2_ at 37 °C.

### Patients and Tissue Samples

A TMA containing 90 HCC and 90 matched non‐tumor tissues was provided by the National Center for Biochip at Shanghai (HLivH180Su16, Shanghai, China). Twelve pairs of fresh primary HCC and corresponding adjacent non‐tumor tissues were collected and immediately frozen in liquid nitrogen after patients with HCC underwent radical hepatectomy at Zhejiang Provincial People's Hospital (Hangzhou, China). None of the patients received local or systemic therapy before surgery. Informed consents were obtained from all patients, and all experimental procedures were approved by the Ethics Committee of Zhejiang Provincial People's Hospital (KT2022041).

### Plasmids and Transient Transfection

Overexpression plasmids containing HA‐MAP4K4, Flag‐G3BP2, and Flag‐G3BP2‐T227A were constructed and purchased from Hanbio Biotechnology, Inc. (Shanghai, China). For transient expression, HEK293T or HCC cells were transfected with plasmids using Lipofectamine 2000 (11 668 030; Invitrogen, Carlsbad, CA, USA) according to the manufacturer's instructions. Briefly, HEK293T and HCC (Huh7 and PLC/PRF/5) cells were seeded in 6 cm culture dish (2.5 × 10^6^ cells per dish) and 6‐well plates (1.0 × 10^6^ cells per well). At the confluence of 80%, cells were transfected with the mixture of plasmid and transfection reagent (1 µg: 2 µL) for 24 h and then replaced with fresh culture medium for another 24 h. In the 6‐cm culture dish, the total amount of plasmids was 5 µg, whereas the total amount was 2 µg in the 6‐well plate. The total number of plasmids in a specific dish or plate was divided equally for each plasmid.

### Lentiviral‐Infected Stable Cell Lines

The lentiviral short hairpin RNAs (shRNAs) against PEPT1 (shPEPT1‐1: GCAGTCACCTCAGTAAGCTCCATTA, shPEPT1‐2: CACGGGATTGGAATTCTCATATTCT), MAP4K4 (shMAP4K4‐1: GGGAAGGTCTATCCTCTTA, shMAP4K4‐2: TAAGTTACGTGTCTACTAT), G3BP2 (shG3BP2‐1: TGAAGGATCTGTTCCAAATAA, shG3BP2‐2: CGGGAGTTTGTGAGGCAATAT), or scramble control were obtained from Tsingke Biotechnology Co., Ltd. (Beijing, China). Lentiviral particles were produced in HEK293T cells, according to the manufacturer's instructions (Inovogen Tech. Co., Beijing, China). After transfection for 48 h, lentiviral particles were collected, and HCC cells were infected with 8 µg mL^−1^ polybrene. Stable cells were selected after transfection with the lentivirus for 72 h using 2 µg mL^−1^ puromycin (InvivoGen) for 1 week.

### Western Blotting

Immunoblotting was performed as previously described.^[^ ^24^ ^]^ Briefly, the cells were collected and lysed on ice using radioimmunoprecipitation assay RIPA lysis buffer (Beyotime, Shanghai, China). After quantification using a BCA protein assay kit (Beyotime), lysates were separated using 10% sodium dodecyl sulfate‐polyacrylamide gel electrophoresis (SDS‐PAGE) and transferred to polyvinylidene difluoride membranes (Millipore, MO, USA). Membranes were blocked with 5% non‐fat milk (Bio‐Rad) for 1 h at room temperature and then incubated overnight at 4 °C with the following primary antibodies: PEPT1 (1:1000, Huabio, #HA500444), MAP4K4 (1:1000, Cusabio, #CSB‐PA013439LA01HU), G3BP2 (1:1000, Proteintech, #16276‐1‐AP), phospho‐Threonine (1:1000, CST, #9386), E‐cadherin (1:1000, Proteintech, #20874‐1‐AP), N‐cadherin (1:1000, Proteintech, #22018‐1‐AP), vimentin (1:2500, Proteintech, #10366‐1‐AP), β‐catenin (1:1000, Huabio, #HA500444), and β‐actin (1:10 000, Proteintech, #66009‐1‐Ig). Next, the membranes were incubated with horseradish peroxidase‐conjugated rabbit or mouse secondary antibodies, and immunoreactive signals were detected by adding enhanced chemiluminescence substrate. Subsequently, the images were visualized using the ChemiDoc Imaging System (Bio‐Rad) and analyzed using the ImageJ software (NIH, Wayne Rasband, USA; V.1.6.0).

### Co‐IP Assay

HEK293T cells were seeded and transfected with the indicated plasmids. Total protein was extracted using a co‐IP (Beyotime) lysis buffer with protease inhibitors (Beyotime). The lysates were incubated with anti‐HA (ABclonal, #AE008) antibody, protein A/G magnetic beads (Selleck, Houston, TX, USA) or anti‐FLAG magnetic beads (Selleck) for immunoprecipitation at 4 °C on a rotator overnight. After incubation, the beads were pelleted and washed five times with TBS (50 mM Tris‐HCl and 150 mm NaCl; pH 7.4). Finally, the immunoprecipitates were eluted with 1 × SDS‐PAGE loading buffer and collected for Western blot analysis. For endogenous co‐IP, lysates were incubated with anti‐MAP4K4 (Proteintech, #55247‐1‐AP) or anti‐G3BP2 (Proteintech, #16276‐1‐AP) antibodies or negative control IgG (CST, #2729) overnight, followed by conjugation with protein A/G magnetic beads for an additional 4 h. After incubation, the beads were pelleted and washed five times with TBS (50 mm Tris‐HCl, 150 mM NaCl, pH 7.4), and the immunoprecipitates were collected for Western blot analysis.

### Immunofluorescence Assay

The immunofluorescence assay was performed as described previously.^[^ ^24^ ^]^ Briefly, after the indicated treatments, the cells were cultured in a 15 mm glass‐bottom confocal dish for 24 h. The cells were then washed with phosphate buffered saline (PBS), fixed with 4% paraformaldehyde for 15 min, and permeabilized with 0.5% Triton X‐100 for 20 min. After blocking, the slides were staining overnight at 4 °C with indicated primary antibodies: HA (1:50, Proteintech, #51064‐2‐AP), Flag (1:2000, Proteintech, #66008‐4‐Ig), and MAP4K4 (1:50, Cusabio, #CSB‐PA013439LA01HU). The fixed cells were washed thrice and incubated with fluorescent secondary antibodies (iFluor 488‐conjugated goat anti‐Mouse IgG and iFluor 594‐conjugated goat anti‐Rabbit IgG, 1:400, Huabio) for 2 h. Finally, DAPI (Beyotime, Shanghai, China) was used to stain the cell nuclei for 5 min before visualization by confocal microscopy (Leica, TCS SP8).

### Wound Healing Assay

HCC cells were seeded in a 24‐well plate and grown to a confluent monolayer of cells, and a micropipette tip was used to create a cross‐wound. After scratching, the cells were washed thrice with PBS and photographed at the time point as 0 h. The cells were cultured in serum‐free DMEM or RPMI‐1640 for 24 h and photographed at this time point. The relative migration was calculated by measuring the width of the wounds at 0 and 24 h.

### Transwell Assay

Cell migration and invasion assays were performed using 24‐well Transwell chambers (8 µm pore size, Corning, Cat. 8432, USA), as previously described.^[^ ^47^ ^]^ For the invasion assay, the upper chamber was pre‐coated with 250 µg mL^−1^ Matrigel (BD Bioscience, Cat. 356 234, USA) and incubated at 37 °C for 2 h. For the Transwell assay, approximately 3 × 10^4^ of Bel7405, HCCLM3, Huh7, or PLC/PRF/5 cells were suspended in 200 µL serum‐free culture medium and added to the inside of the chamber. Medium containing 10% FBS was added outside the chamber. After incubation for 24 h, the invading cells were washed with PBS, fixed with 4% polyformaldehyde, and stained with 0.1% crystal violet. Non‐invading cells on the upper side of the chamber were erased using a cotton swab. More than three microscopic fields were randomly selected from each chamber to determine the average number of cells.

### Treatment with MAP4K4 Inhibitor

Huh7 and PLC/PRF/5 cells were treated with the MAP4K4 inhibitor GNE‐495 (MedChemExpress, Shanghai, China) or DMSO to examine its effects on cell proliferation, migration, and invasion in vitro. For the cell proliferation assay, cells were seeded in 96‐well plates and treated with different concentrations of GNE‐495 (0, 0.2, 0.5, 1.0, 2.0, 5.0, and 10.0 µm) for 24 h. Then, cell proliferation was determined according to the manufacturer's protocol of CCK8 (Biosharp). For the Western blot assay, cells were seeded in 6‐well plates and treated with different concentrations of GNE‐495 (0, 0.2, 0.5, 1.0, 2.0, 5.0, and 10.0 µm) for 24 h. Then, cells were collected and further analyzed via Western blot. For the cell migration and invasion assay, cells were cultured on the upper side of the chamber and treated with 2 µm GNE‐495 for 24 h. Then, the cells were analyzed using a Transwell assay.

### Mass Spectrometry (MS) Analysis

For proteomic analysis, PEPT1‐knockdown and control Huh7 cells were collected and immediately frozen in liquid nitrogen. LC‐MS/MS analysis of the samples to identify proteins interacting with PEPT1 was determined by BIOZON Medical Laboratory Co., Ltd. (Hangzhou, China). For phosphorylation proteomic analysis, MAP4K4‐knockdown and control Huh7 cells were subjected to the procedures described above. To validate the proteins interacting with MAP4K4 and their phosphorylated sites, MS analysis was performed using iProteome Biotechnology Co., Ltd. (Shanghai, China).

### IHC Analysis

Paraffin‐embedded tissue sections were deparaffinized, hydrated, and soaked in 3% H_2_O_2_ for 20 min at room temperature and subsequently incubated with anti‐PEPT1 (1:400, Huabio, #HA500444), anti‐MAP4K4 (1:100, Proteintech, #55247‐1‐AP), and anti‐G3BP2 (1:2400, Proteintech, #16276‐1‐AP) antibodies at 4 °C overnight. The slides were then incubated with horseradish peroxidase‐conjugated goat anti‐rabbit antibody for 1 h, stained with diaminobenzidine (DAB, Beyotime, Hangzhou, China), and subsequently counterstained with hematoxylin. IHC staining was evaluated by two clinical pathologists who were blinded to patient characteristics and clinical outcomes. Scoring was based on both the extent and intensity of the staining. The staining extent score, based on the percentage of positively stained cells, was divided into five levels: 0 (< 10%), 1 (10–25%), 2 (26–50%), 3 (51–75%), and 4 (> 75%). The staining intensity score was divided into four levels: 0 (negative staining), 1 (weak staining), 2 (moderate staining), and 3 (strong staining). The final IHC score was calculated by multiplying the staining extent with the intensity scores. Specimens with final scores ranging from 0 to 6 were defined as the low‐expression group, whereas those with final scores ranging from 7 to 12 were defined as the high‐expression group.

### In Vivo Animal Study

Animal studies compiled with ethical regulations for animal testing and research and were approved by and performed in accordance with Institutional Animal Care and Use Committee of Zhejiang Provincial People's Hospital (A20220069). For lung metastasis, HCC cells (1 × 10^6^) were injected into the tail vein of each BALB/c nude mice (4‐week old mice). Six weeks post‐injection, the mice were intraperitoneally injected with D‐luciferin (7.5 mg mL^−1^, 100 µL per mouse). They were anesthetized using isoflurane inhalation and photographed using an IVIS imaging system (Xenogen). After bioluminescence imaging, all mice were euthanized, and lungs were removed and fixed with 4% paraformaldehyde at 4 °C overnight. The intact lungs were then embedded in paraffin and sectioned for H&E or IHC staining. The focus numbers on the surface of the lungs were counted using a microscope, followed by standard H&E staining.

### Non‐Targeted Metabolomics Study

PEPT1‐knockdown and control Huh7 cells were collected, and a precooled 80% methanol aqueous solution was added to an ultrasonic homogenizer to break up the cells. An internal standard metabolite was added to the mixture and extracted via ultrasonication, and the supernatant of the extract was dried. Finally, the samples were redissolved in 20% methanol and subjected to LC‐MS/MS analysis (Oebiotech, Shanghai, China).

### Pro‐Gly Uptake Study

Pro‐Gly uptake study was performed using a previously described method.^[^ ^48^ ^]^ First, confluent monolayer of HCC cells was washed twice with MES buffer (8 g NaCl, 0.4 g KCl, 0.14 g CaCl_2_, 0.2 g MgSO_4_·7H_2_O, 0.06 g Na_2_HPO_4_·12H_2_O, 0.06 g KH_2_PO_4_, 0.35 g NaHCO_3_, 1 g glucose, 1.95 g MES in 1 L ddH_2_O, pH 6.5) at 37 °C. The cells were then incubated with MES buffer containing 100 µm Pro‐Gly for 1 h. The uptake study was terminated by removing the buffer and immediately washing the cells thrice with ice‐cold MES buffer. The cells were then treated with 0.1% Triton X‐100, and the concentrations were determined using HPLC‐MS/MS (AB SCIEX 4500, AB SCIEX, Framingham, MA, USA).

### Statistical Analyses

Statistical analysis and graphics generation were performed using GraphPad Prism Version 8.0. All data were presented as mean ± standard deviation (SD). Differences between two groups were compared using a two‐tailed Student's t‐test. Comparisons between more than two groups were performed using one‐way analysis of variance with follow‐up Dunnett T3 tests. *P* < 0.05 was considered statistically significant.

## Conflict of Interest

The authors declare no conflict of interest.

## Author Contributions

F.S., Z.Z., and W.L contributed equally to this work. F.S., Y.Z., and P.H. conceived and designed the study. F.S., Z.Z., T.X., Q.W., W.Z., L.G., Q.H., and S. Z. performed the experiments, developed the methodology, and wrote, reviewed, and revised the manuscript. F.S., Z.Z., X.H., and W.Z. performed the acquisition, analysis, and interpretation of the data and statistical analysis. F.S., W.L., C.Z., X.R., and W.F. provided technical and material support. All the authors have read and approved the final version of this manuscript.

## Supporting information

Supporting Information

## Data Availability

The data that support the findings of this study are available from the corresponding author upon reasonable request.
