# Correction to “Peptide Transporter 1‐Mediated Dipeptide Transport Promotes Hepatocellular Carcinoma Metastasis by Activating MAP4K4/G3BP2 Signaling Axis”

**DOI:** 10.1002/advs.202504116

**Published:** 2025-04-26

*Adv. Sci*. **2024**, 1, 2306671.

https://doi.org/10.1002/advs.202306671

We found the bands of β‐actin in Figure 4A were misused. The corrected Figure 4A is shown as below. This correction does not affect any conclusions of the paper.

Corrected Figure 4A


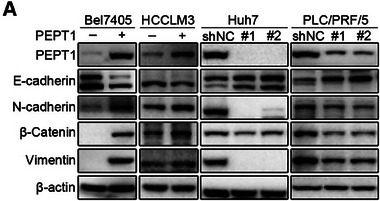


We apologize for this error.

