# Supplementary material for: Peptide Transporter 1‐Mediated Dipeptide Transport Promotes Hepatocellular Carcinoma Metastasis by Activating MAP4K4/G3BP2 Signaling Axis
Source: Adv Sci (Weinh). 2024 Apr 19;11(24):2306671. doi: 10.1002/advs.202306671 (PMC11200092; doi:10.1002/advs.202306671)
Supplement: Supplementary file 1 — Supporting Information [file ADVS-11-2306671-s001.pdf]

ADVANCED SCIENCE
Open Access

Supporting Information

for *Adv. Sci.*, DOI 10.1002/advs.202306671

Peptide Transporter 1-Mediated Dipeptide Transport Promotes Hepatocellular Carcinoma Metastasis by Activating MAP4K4/G3BP2 Signaling Axis

Feifeng Song, Zhentao Zhang, Weifeng Liu, Tong Xu, Xiaoping Hu, Qiyue Wang, Wanli Zhang, Luqi Ge, Chengwu Zhang, Qing Hu, Hui Qin, Song Zhang, Xinxin Ren, Weijiao Fan, Yiwen Zhang and Ping Huang**

Supporting Information

Peptide Transporter 1-mediated Dipeptide Transport Promotes Hepatocellular Carcinoma Metastasis by Activating MAP4K4/G3BP2 Signaling Axis

Feifeng Song, Zhentao Zhang, Weifeng Liu, Tong Xu, Xiaoping Hu, Qiyue Wang, Wanli Zhang, Luqi Ge, Chengwu Zhang, Qing Hu, Hui Qin, Song Zhang, Xinxin Ren, Weijiao Fan, Yiwen Zhang, and Ping Huang**

Supporting Information

Title: Peptide Transporter 1-mediated Dipeptide Transport Promotes Hepatocellular Carcinoma Metastasis by Activating MAP4K4/G3BP2 Signaling Axis

Feifeng Song†, Zhentao Zhang†, Weifeng Liu†, Tong Xu, Xiaoping Hu, Qiyue Wang, Wanli Zhang, Luqi Ge, Chengwu Zhang, Qing Hu, Hui Qin, Song Zhang, Xinxin Ren, Weijiao Fan, Yiwen Zhang*, Ping Huang*

Content

Supplementary Figures (Figure S1-S6)

Supplementary Tables (Table S1-S4)

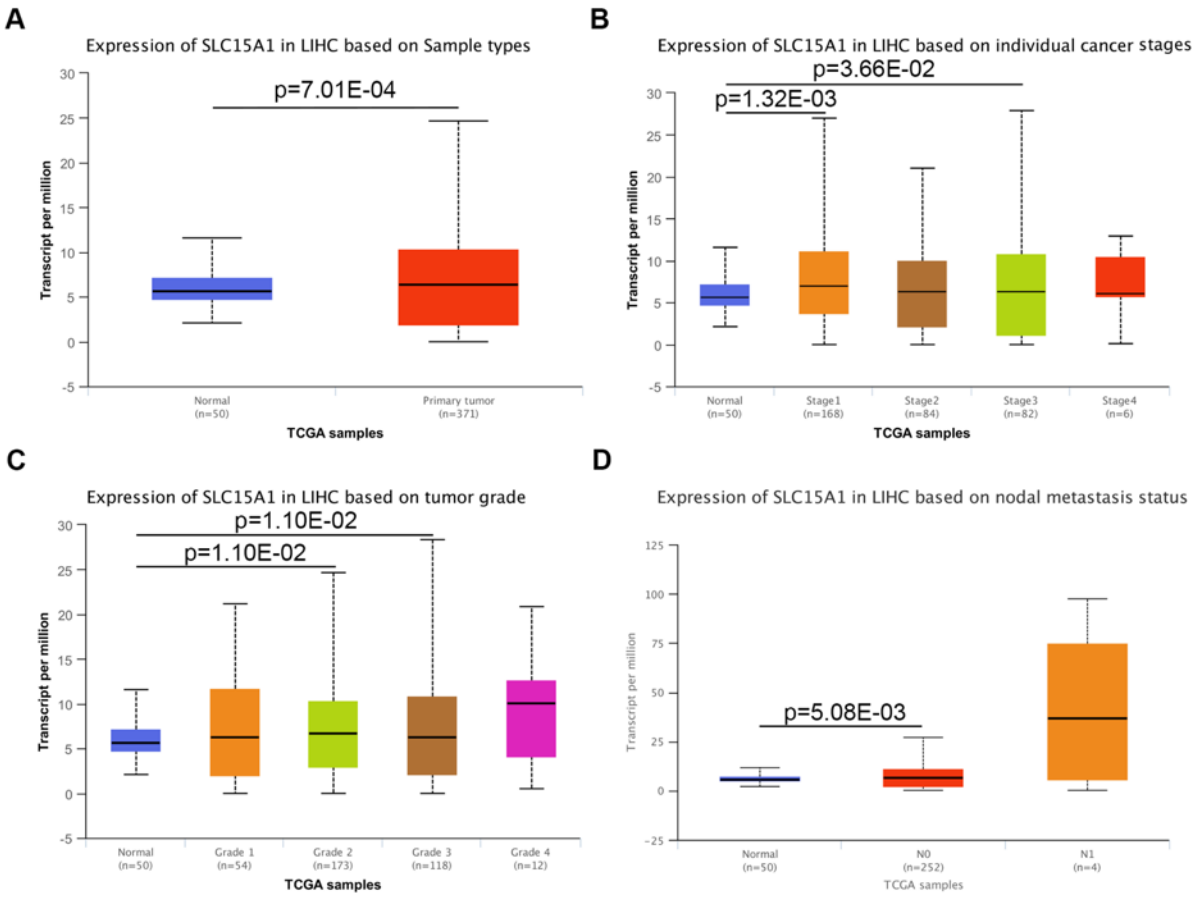

Supplementary Figure S1. PEPT1 (SLC15A1) expression in HCC based on sample types (A) and tumor stages (B), tumor grade (C) and nodal metastasis status (D) was conducted by UALCAN platform.

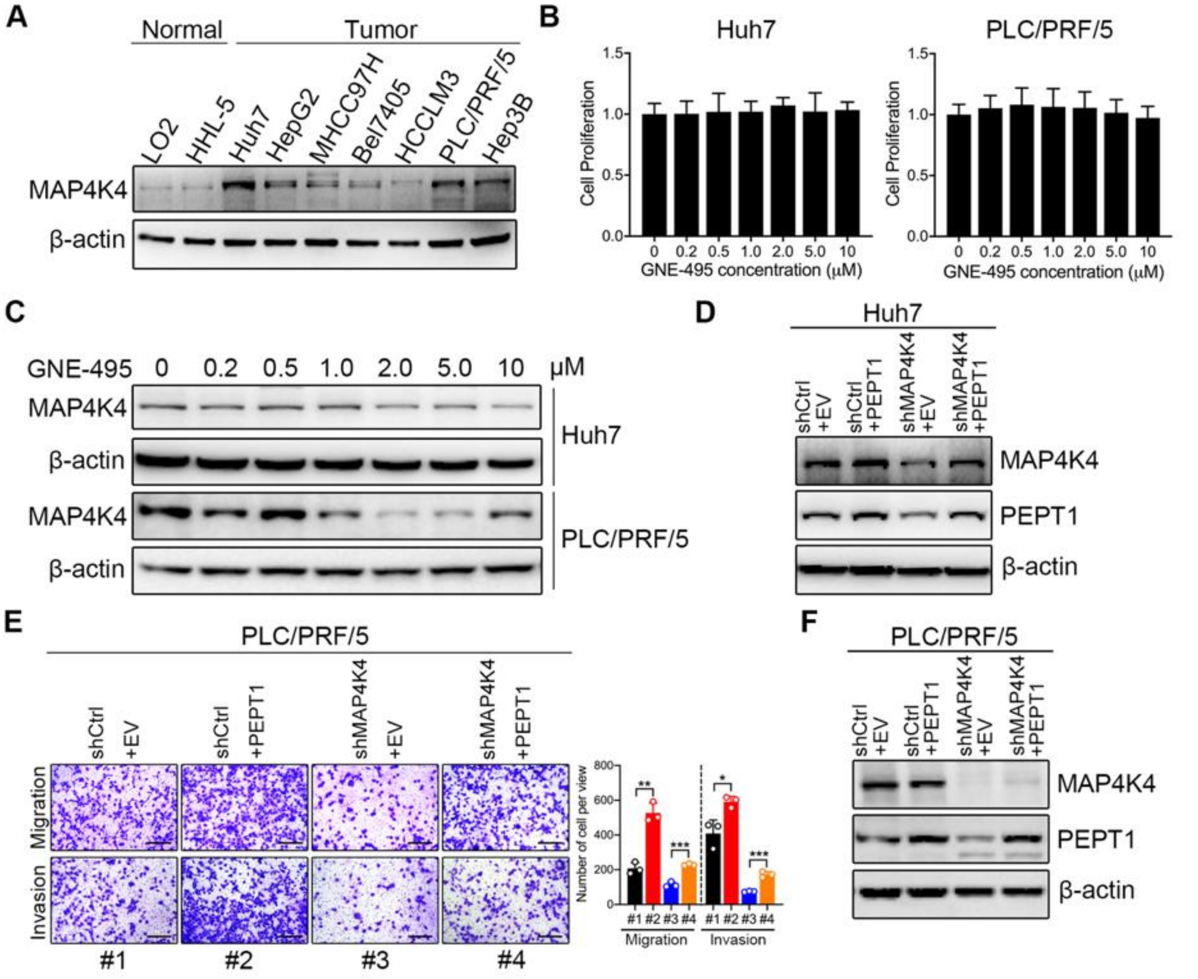

Supplementary Figure S2. (A) Expression of MAP4K4 in hepatocytes and several hepatocellular carcinoma cell lines. (B) Huh7 and PLC/PRF/5 cells were treated with different concentration MAP4K4 inhibitor (GNE-495) for 24 hours. Cell proliferation ability was determined by CCK8 assay. (C) Huh7 and PLC/PRF/5 cells were treated with different concentrations of GNE-495 for 24 hours and detected the protein expression of MAP4K4 by Western blotting. (D) MAP4K4-knockdown Huh7 cells were transfected with PEPT1 plasmid for 48 hours, and detected the expression of MAP4K4 and PEPT1 by Western blotting. (E) MAP4K4-knockdown PLC/PRF/5 cells were transfected with PEPT1 for 48 hours. The protein expression of PEPT1 and MAP4K4 was detected by Western blotting (F) and the cell migration and invasion were detected by Transwell assay (E). Scale bar, 250 μm.*, $P < 0.05$; **, $P < 0.01$; and ***, $P < 0.001$.

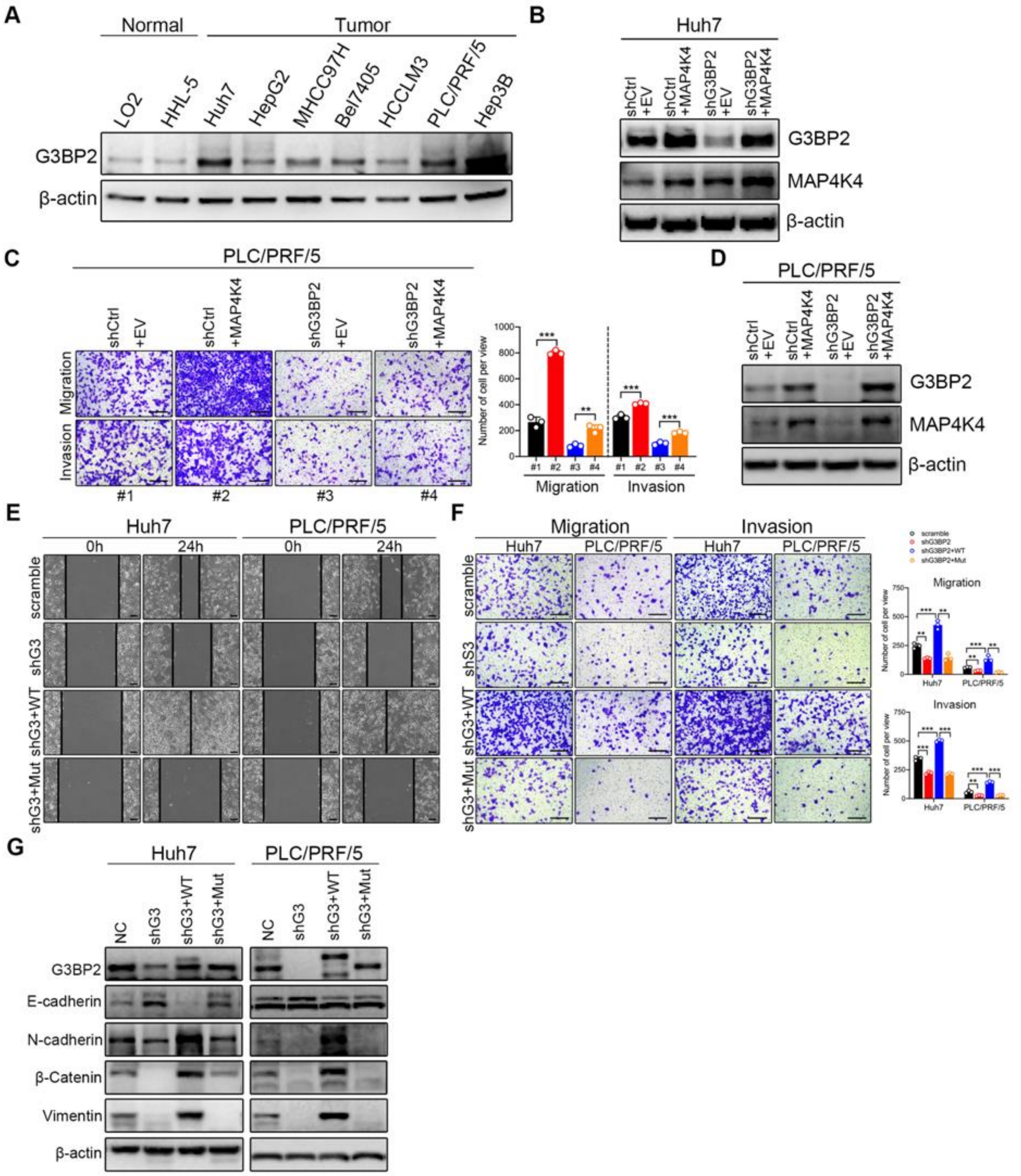

Supplementary Figure S3. (A) Expression of G3BP2 in hepatocytes and several hepatocellular carcinoma cell lines. (B) G3BP2-knockdown Huh7 cells were transfected with MAP4K4 plasmid for 48 hours, and detected the expression of G3BP2 and MAP4K4 by Western blotting. (C-D) G3BP2-knockdown PLC/PRF/5 cells were transfected with MAP4K4 for 48 hours. The protein expression of G3BP2 and MAP4K4 was detected by Western blotting (C) and the cell migration and invasion were detected by Transwell assay (D). Scale bar, 250

μm. (E) Representative images and quantification of the indicated cells in the wound-healing migration assays. Scale bar, 100 μm. F) Representative images and quantification of the migration and invasion of the indicated cells in the Transwell assays. Scale bar, 250 μm. G) Protein expression of EMT-associated proteins in HCC cells with G3BP2 knockdown or G3BP2 wildtype (mutant) overexpression. Scale bar, 250 μm. *, $P < 0.05$; **, $P < 0.01$; and ***, $P < 0.001$.

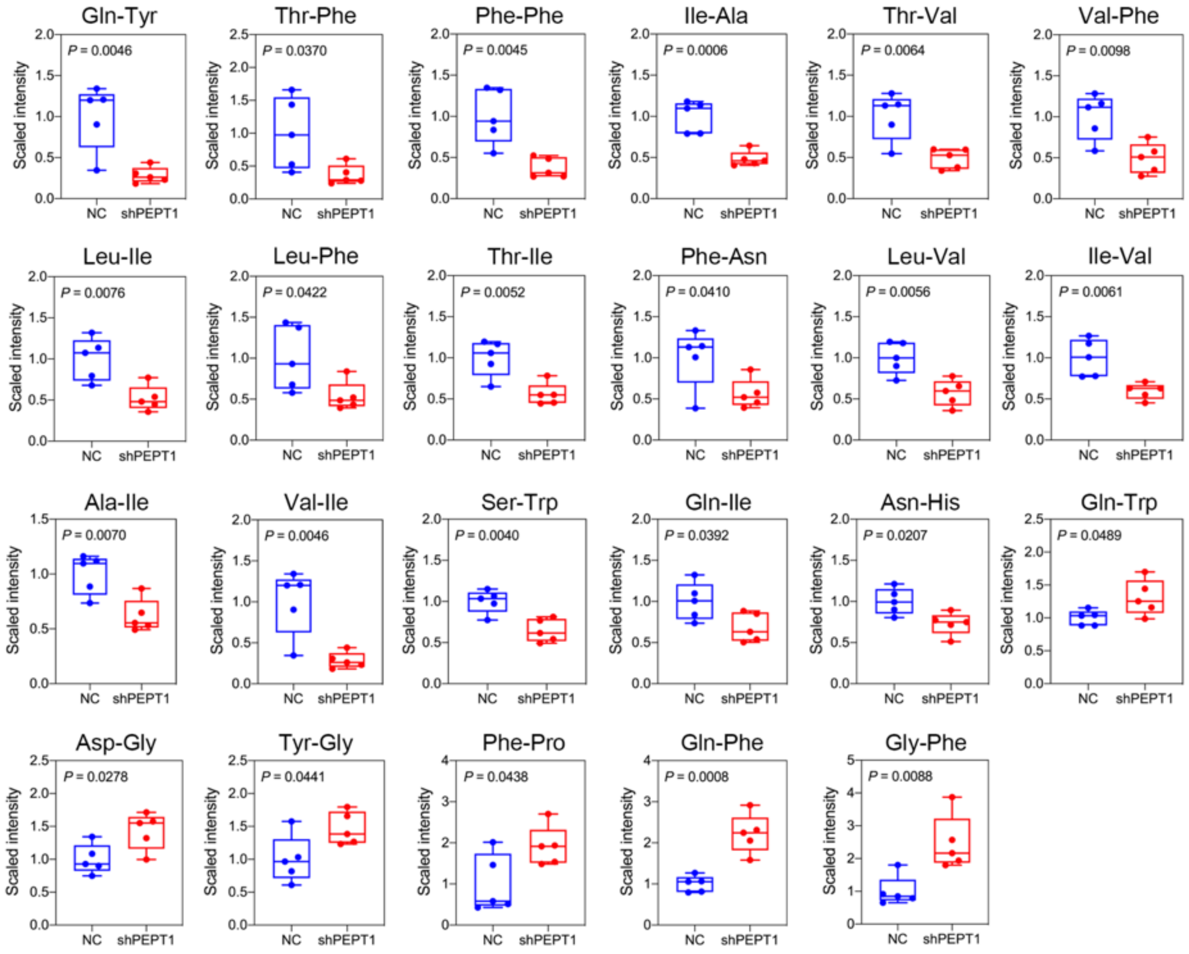

Supplementary Figure S4. Metabolomic analyses of dipeptides in PEPT1-knockdown and control Huh7 cells. Amounts of dipeptides in PEPT1-knockdown and control cells were plotted in whisker boxes. Horizontal line across box, median value; error bars, maximum and minimum of distribution; dot, each data point. *P* value indicates the statistical significance among PEPT1-knockdown versus control cells as measured by unpaired *t*-test.

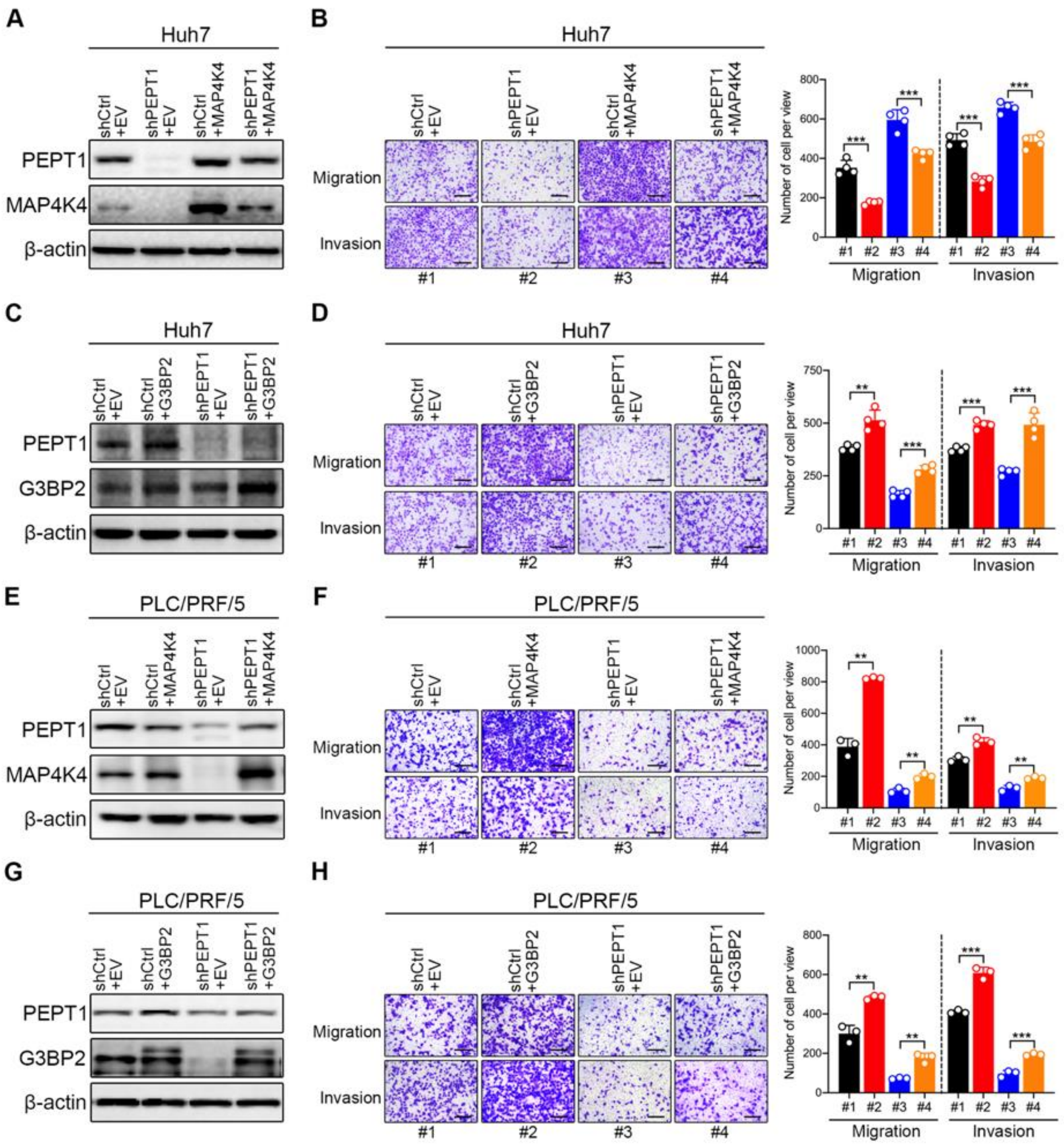

Supplementary Figure S5. (A-B, E-F) PEPT1-knockdown Huh7 (A-B) and PLC/PRF/5 (E-F) cells were transfected with MAP4K4 for 48 hours. The protein expression of PEPT1 and MAP4K4 was detected by Western blotting (A, E) and the cell migration and invasion were detected by Transwell assay (B, F). (C-D, G-H) PEPT1-knockdown Huh7 (C-D) and PLC/PRF/5 (G-H) cells were transfected with G3BP2 for 48 hours. The protein expression of G3BP2 and PEPT1 was detected by Western blotting (C, G) and the cell migration and invasion were detected by Transwell assay (D, H).

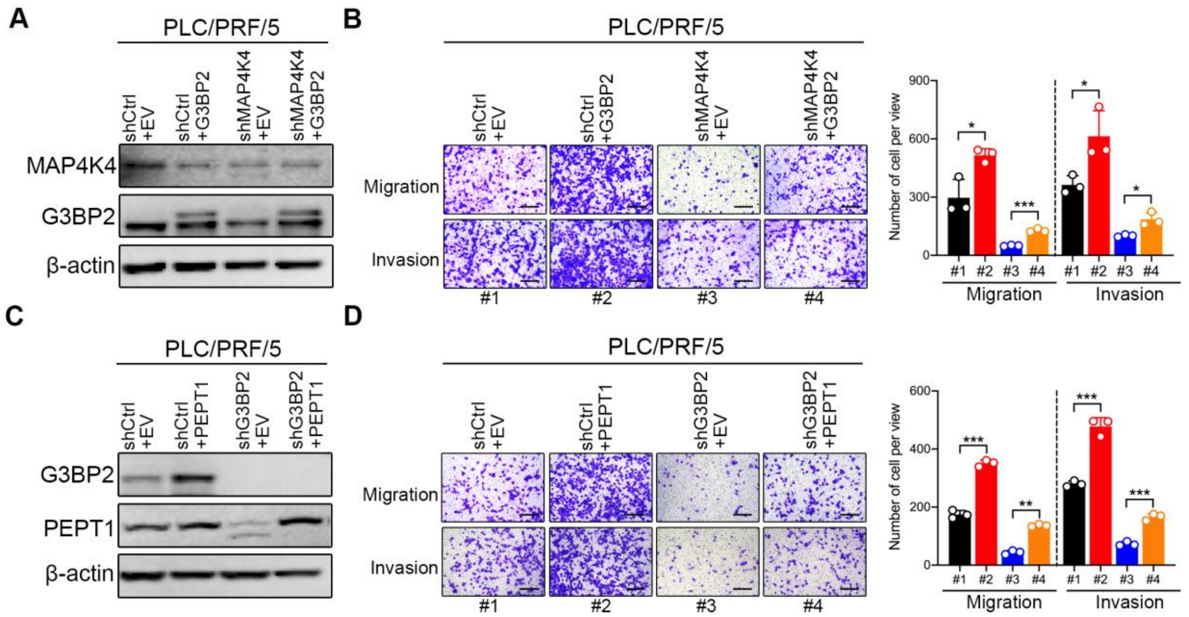

Supplementary Figure S6. (A-B) MAP4K4-knockdown PLC/PRF/5 cells were transfected with G3BP2 for 48 hours. The protein expression of MAP4K4 and G3BP2 was detected by Western blotting (A) and the cell migration and invasion were detected by Transwell assay (B). (C-D) G3BP2-knockdown PLC/PRF/5 cells were transfected with PEPT1 for 48 hours. The protein expression of PEPT1 and G3BP2 was detected by Western blotting (C) and the cell migration and invasion were detected by Transwell assay (D).

Table S1. Clinicopathological characteristics of patients with hepatocellular carcinoma.

Variables	No. of patients	Percentage (%)
Sex		
Male	80	88.9
Female	10	11.1
Age(years)		
≤ 50	40	44.4
> 50	50	55.6
ALT (U/L)*		
≤ 40	52	58.4
> 40	37	41.6
Serum AFP (g/L)*		
< 400	57	64.0
≥ 400	32	36.0
GGT(U/L)*		
≤ 54	51	57.3
> 54	38	42.7
HBV*		
Negative	9	10.1
Positive	80	89.9
HCV*		
Negative	88	98.9
Positive	1	1.1
Tumor number		
Single	79	87.8
Multiple	11	12.2
Tumor size (cm)		
< 5	55	61.1
≥ 5	35	38.8
Tumor encapsulation*		
Present	42	47.2
Absent	47	52.8

Liver cirrhosis nodules*		
≤ 1	9	10.1
>1	80	89.9
TNM stage		
T1	63	70.0
T2+T3	27	30.0
Recurrence		
Yes	49	54.4
No	41	45.6

*: One missing data.

Table S2. Clinicopathological characteristics and staining patterns of PEPT1 in HCC

Variables	Total	PEPT1 expression				P value
		High (33)		Low (57)		
	90	No. of patients	%	No. of patients	%	
Sex						
Male	80	31	38.8	49	61.2	0.3
Female	10	2	20.0	8	80.0	
Age (years)						
≤ 50	40	12	30.0	28	70.0	0.2
> 50	50	21	42.0	29	58.0	
ALT (U/L)*						
≤ 40	52	20	38.5	32	61.5	0.6
> 40	37	12	32.4	25	67.6	
Serum AFP (g/L)*						
< 400	57	26	45.6	31	54.4	0.011
≥ 400	32	6	18.8	26	81.2	
GGT (U/L)*						
≤ 54	51	16	31.4	35	68.6	0.3
> 54	38	16	42.1	22	57.9	
HBV*						
Negative	6	2	33.3	4	66.7	> 0.9
Positive	83	30	36.1	53	63.9	
HCV*						
Negative	88	32	36.4	54	61.4	> 0.9
Positive	1	0	0	1	100	
Tumor number						
Single	79	27	34.2	52	65.8	0.2
Multiple	11	6	54.5	5	45.5	
Tumor size (cm)[#]						
< 5	60	22	36.7	38	63.3	> 0.9
≥ 5	30	11	36.7	19	63.3	
Tumor encapsulation*						
Present	42	15	35.7	27	64.3	> 0.9

Absent	47	17	36.2	30	63.8	
Liver cirrhosis nodules*						
≤ 1	9	2	22.2	7	77.8	0.5
>1	80	30	37.5	50	62.5	
TNM stage						
T1	63	22	34.9	41	65.1	0.6
T2+T3	27	11	40.7	16	59.3	
Recurrence						
Yes	49	22	44.9	27	55.1	0.076
No	41	11	26.8	30	73.2	
PEPT1						
Low	57					
High	33					

* One missing data.

[#] Tumor size was measured by the length of the largest tumor nodule.

P value: Fisher's exact test.

Table S3. Univariate analysis for survival and recurrence in HCC patients.

Variable	Survival			Recurrence		
	HR	95% CI	*P*	HR	95% CI	*P*
Sex						
Male vs. female	3.7	0.5-27	0.2	1.3	0.48-3.7	0.58
Age (years)						
> 50 vs. ≤ 50	1.3	0.64-2.7	0.46	1.6	0.88-2.8	0.13
ALT (U/L)						
> 40 vs. ≤ 40	1.4	0.67-2.8	0.38	1.2	0.65-2.1	0.62
Serum AFP (g/L)						
≥ 400 vs. < 400	1.1	0.5-2.2	0.88	0.97	0.53-1.8	0.92
GGT (U/L)						
> 54 vs. ≤ 54	1.7	0.82-3.4	0.16	1.7	0.95-3.0	0.072
HBV						
Positive vs. negative	0.71	0.21-2.4	0.57	0.89	0.32-2.5	0.82
HCV						
Positive vs. negative	1.1e-7	0-Inf	1.00	1.1e-7	0-Inf	1.00
Tumor number						
Single vs. multiple	0.72	0.25-2.1	0.55	0.52	0.23-1.2	0.11
Tumor size (cm)						
≥ 5 vs. < 5	1.9	0.9-3.8	0.093	1.8	0.98-3.2	0.057
Tumor encapsulation						
Present vs. absent	0.53	0.25-1.1	0.097	0.74	0.41-1.3	0.3
Liver cirrhosis nodules						
> 1 vs. ≤ 1	3.8	0.51-28	0.19	1.4	0.51-3.9	0.51
TNM stage						
T1 vs. T2+T3	2.2	1.1-4.6	0.029	1.5	0.84-2.8	0.17
PEPT1 expression						
Low vs. high	0.43	0.21-0.89	0.022	0.57	0.32-1.0	0.058

Table S4. 5 proteins might interact with MAP4K captured by phosphorylation proteomic analysis

No.	Protein	Peptide	*P* value	Phosphosites
1	APC	SGRSPtGNTPPVIDSVSEK	0.00222	T2676
2	ATF2	NDSVIVADQTPtPTR	0.01052	T71
3	CABLES1	NtIDSTSSFSQFR	0.01861	T415
4	G3BP2	STtPPPAEPVSLPQEPPK	0.00903	T227
5	PRDM2	KPSQtLQPSEDLADGK	0.0429	T423
